# Different Types of Atrial Fibrillation Share Patterns of Gut Microbiota Dysbiosis

**DOI:** 10.1128/mSphere.00071-20

**Published:** 2020-03-18

**Authors:** Kun Zuo, Xiandong Yin, Kuibao Li, Jing Zhang, Pan Wang, Jie Jiao, Zheng Liu, Xiaoqing Liu, Jiapeng Liu, Jing Li, Xinchun Yang

**Affiliations:** a Heart Center & Beijing Key Laboratory of Hypertension, Beijing Chaoyang Hospital, Capital Medical University, Beijing, China; b Capital Medical University, Beijing, China; University of Utah

**Keywords:** atrial fibrillation, gut microbiota, metabolomics, metagenomics, paroxysmal, persistent

## Abstract

Dysbiotic gut microbiota (GM) and disordered metabolic patterns are known to be involved in the clinical expression of atrial fibrillation (AF). However, little evidence has been reported in characterizing the specific changes in fecal microbiota in paroxysmal AF (PAF) and persistent AF (psAF). To provide a comprehensive understanding of GM dysbiosis in AF types, we assessed the GM signatures of 30 PAF patients, 20 psAF patients, and 50 non-AF controls based on metagenomic and metabolomic analyses. Compared with control subjects, similar changes of GM were identified in PAF and psAF patients, with elevated microbial diversity and similar alteration in the microbiota composition. PAF and psAF patients shared the majority of differential taxa compared with non-AF controls. Moreover, the similarity was also illuminated in microbial function and associated metabolic alterations. Additionally, minor disparity was observed in PAF compared with psAF. Several distinctive taxa between PAF and psAF were correlated with certain metabolites and atrial diameter, which might play a role in the pathogenesis of atrial remodeling. Our findings characterized the presence of many common features in GM shared by PAF and psAF, which occurred at the self-terminating PAF. Preventative and therapeutic measures targeting GM for early intervention to postpone the progression of AF are highly warranted.

**IMPORTANCE** Atrial fibrillation has been identified to be associated with disordered gut microbiota. Notably, atrial fibrillation is a progressive disease and could be categorized as paroxysmal and persistent based on the duration of the episodes. The persistent atrial fibrillation patients are accompanied by higher risk of stroke and lower success rate of rhythm control. However, the microbial signatures of different categories of atrial fibrillation patients remain unknown. We sought to determine whether disordered gut microbiota occurs in the self-terminating PAF or intestinal flora develops dynamically during atrial fibrillation progression. We found that different types of atrial fibrillation show a limited degree of gut microbiota shift. Gut microbiota dysbiosis has already occurred in mild stages of atrial fibrillation, which might act as an early modulator of disease, and therefore may be regarded as a potential target to postpone atrial fibrillation progression.

## Abstract (precis)

Atrial fibrillation has been identified to be associated with disordered gut microbiota. Notably, atrial fibrillation is a progressive disease and could be categorized as paroxysmal and persistent based on the duration of the episodes. The persistent atrial fibrillation patients are accompanied by higher risk of stroke and lower success rate of rhythm control. However, the microbial signatures of different categories of atrial fibrillation patients remain unknown. We sought to determine whether disordered gut microbiota occurs in the self-terminating PAF or intestinal flora develops dynamically during atrial fibrillation progression. We found that different types of atrial fibrillation show a limited degree of gut microbiota shift. Gut microbiota dysbiosis has already occurred in mild stages of atrial fibrillation, which might act as an early modulator of disease, and therefore may be regarded as a potential target to postpone atrial fibrillation progression.

## INTRODUCTION

Atrial fibrillation (AF), one of the most common arrhythmias with heavy global burden, affects approximately 3% of the adult population and almost 6% of persons older than 65 years (1). AF patients experience a variety of symptoms including palpitations, dyspnea, chest tightness, and psychosocial distress, which are caused by the rapid and irregular beating of the atria (2). The presence of AF remarkably increases the risk of stroke and heart failure and decreases the quality and length of human life (2). Notably, AF is a progressive and complicated disease, developing from short, infrequent episodes to longer and more frequent attacks, and many patients develop sustained forms of AF over time (3, 4). These bouts induce atrial electrical and structural remodeling, complex self-sustaining electrical activity, and atrial fibrosis, which contribute to AF maintenance (5, 6). The most commonly used classification of AF relies on the duration of the episodes, which can be classified as paroxysmal (PAF; lasting <7 days) and persistent (psAF; lasting >7 days) (2). The psAF patients are accompanied by more severe symptoms, higher risk of stroke, and lower success rate of rhythm control (7, 8). AF is a progressive disease. The transition from self-terminating paroxysmal state to non-self-terminating persistent AF has been defined as AF progression (9). The detailed mechanisms underlying AF formation and development are largely unknown. Therefore, identifying potential biomarkers for the risk of AF, blocking progression to more severe disease, and performing corresponding treatment should be emphasized.

Recently, with the establishment of the cardiac-gut axis concept (10, 11), increasing evidence suggests that the gut microbiota (GM) plays an important regulatory role during the development of cardiovascular diseases, such as hypertension (HTN) (12, 13), coronary atherosclerotic heart disease (14, 15), heart failure (16), colorectal adenoma-carcinoma (17), etc. Interestingly, the alterations of GM during the progression of diseases could help to further illuminate the role of microbiome in diseases. For example, dysbiosis in the mucosal microbiome was indicative of progression from superficial gastritis, atrophic gastritis, and intestinal metaplasia to gastric cancer (18), as well as from colorectal adenoma to carcinoma (19).

A previous study has identified that alterations in the gut microbial community were correlated with coronary artery disease (CAD) severity via the mediation of serum metabolites (14). Furthermore, the combination of specific bacterial coabundance groups and metabolite modules exhibited potential diagnostic value for differentiating patients with different CAD subtypes (14). Our previous findings have characterized the disordered GM and microbial metabolite profiles in AF patients (20). However, the microbial signatures of different categories of AF patients remain unknown. Whether disordered GM occurs in the self-terminating PAF or intestinal flora develops dynamically during AF progression remains to be explored. These seminal issues encouraged us to identify the patterns of GM in PAF and psAF. To provide a comprehensive understanding of GM dysbiosis in the development of AF, we analyzed the GM and metabolic features in PAF and psAF patients based on high-throughput metagenomic and metabolomic analyses. We identified similar microbial and metabolic profiles with different categories of AF, including PAF and psAF. Our study further established the relationship between gut flora, host metabolomics, and AF severity. Our study reveals that alterations of GM occur at a mild stage of AF, and thus, the manipulation of targeted GM should focus on early prevention of AF in the future.

## RESULTS

### Baseline characteristics of the study cohort.

In the current study, 100 participants consisting of 50 AF patients and 50 non-AF controls (CTRs) were included from our previous study cohort (20). According to AF history and manifestation of cardiogram, AF patients were classified as PAF (*n* = 30) and psAF (*n* = 20) (2). The clinical characteristics of all subjects are shown in Table S1 in the supplemental material. Briefly, the baseline clinical characteristics among the PAF and psAF patients were similar, without statistical difference in age, sex, gender, body mass index (BMI), hypertension, diabetes mellitus, or serum levels of total cholesterol (TC), low-density lipoprotein, fasting blood glucose, creatinine, and glutamic-pyruvic transaminase. Compared to the non-AF CTR group, AF patients were older, presented with higher incidence of type 2 diabetes mellitus (T2DM), lower total cholesterol serum levels, and higher incidence of medications, including angiotensin-converting enzyme inhibitors (ACEIs), angiotensin receptor blockers (ARBs), amiodarone, statins, and dimethyl biguanide (DMBG).

10.1128/mSphere.00071-20.8 TABLE S1 Baseline clinical characteristics of the study cohort. Download Table S1, DOCX file, 0.02 MB. Copyright © 2020 Zuo et al. 2020 Zuo et al. This content is distributed under the terms of the Creative Commons Attribution 4.0 International license.

### Similar gut microbiome diversity between PAF and psAF.

**FIG 1:**
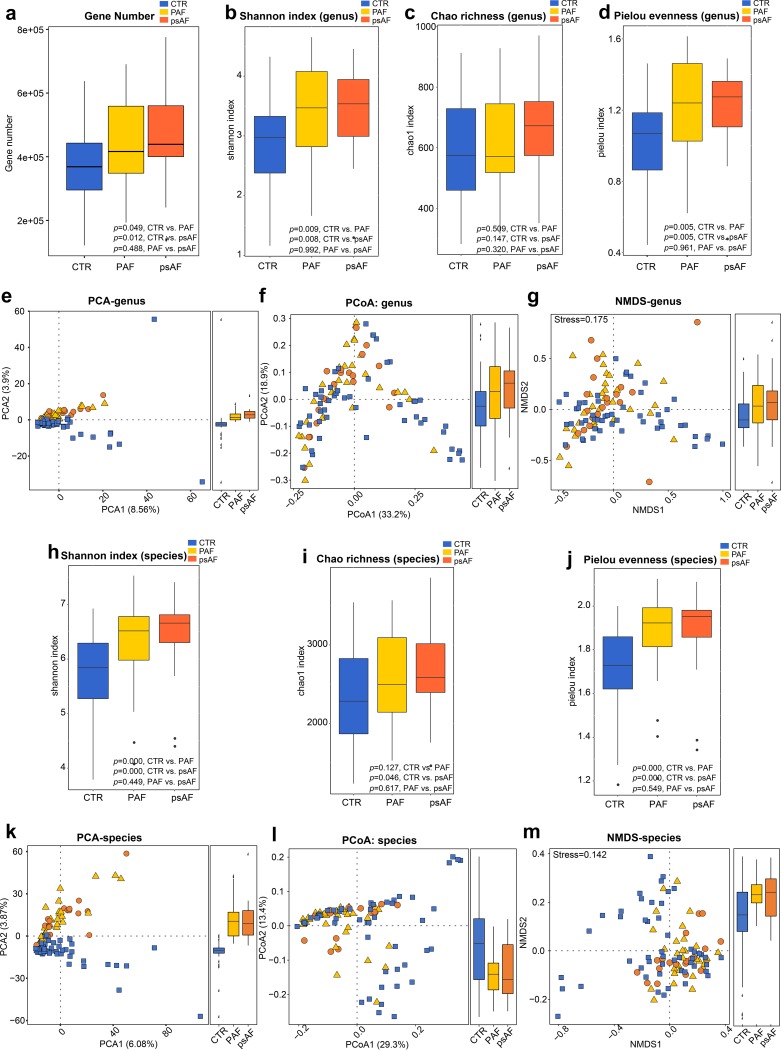
Similar gut microbiome diversity between PAF and psAF. Gene number (a) and within-individual (alpha) diversity including Shannon index at genus level (b) and at species level (h), Chao richness at genus level (c) and at species level (i), Pielou evenness at genus level (d) and at species level (j) in the non-AF control (CTR, blue), paroxysmal atrial fibrillation (PAF, yellow), and persistent atrial fibrillation (psAF, orange). Boxes represent the interquartile ranges, lines inside the boxes denote medians, and circles are outliers. Between-individual (beta) diversity including principal-component analysis (PCA) (e), principal-coordinate analysis (PCoA) (f), nonmetric dimensional scaling (NMDS) (g) based on abundances of the genus is shown in panels e to g, while beta diversity based on species level is shown in panels k to m. The results showed a similar elevated diversity between controls, PAF, and psAF. The blue squares represent non-AF CTR, yellow triangles refer to PAF, and orange circles denote psAF.

Microbial diversity has been considered a biomarker associated with health status and diseases (21). Therefore, both within-individual (alpha) diversity, including Shannon index, Chao richness, Pielou evenness, and gene number, and between-individual (beta) diversity, involving principal-component analysis (PCA), principal-coordinate analysis (PCoA), and nonmetric dimensional scaling (NMDS), were analyzed to assess the GM diversity among different types of AF. At the level of genus and species, we found the alpha diversity parameters were quite similar between PAF and psAF patients (*P* = 0.488 for gene number, Fig. 1a; *P* = 0.992 [genus] and 0.449 [species] for Shannon diversity, Fig. 1b and h; *P* = 0.320 [genus] and 0.617 [species] for Chao richness, Fig. 1c and i; *P* = 0.961 [genus] and 0.549 [species] for Pielou evenness, Fig. 1d and j). No significant discrepancy in GM diversity was observed between PAF and psAF patients. Furthermore, PCA, PCoA, and NMDS analysis showed that AF patients and CTRs clustered into different groups but failed to distinguish different AF patients (*P* > 0.05, analysis of similarity [ANOSIM], PAF versus psAF, Fig. 1e, g, and k to m). These findings indicate that AF patients possess similar microbial features in gut diversity, regardless of whether they manifested clinically as PAF or psAF.

### Similar taxonomic profiles of PAF and psAF.

To evaluate alterations in the microbial structure between non-AF controls and PAF and psAF patients, taxonomic profiles were analyzed. Overall, CTR, PAF, and psAF subjects shared 103 phyla, 90 classes, 181 orders, 368 families, 1,258 genera, and 5,187 species, which occupied the vast majority of microbes annotated in the cohort (Fig. S1 to S3). The top-10 most-abundant genera, such as *Bacteroides*, *Prevotella*, and *Faecalibacterium*, and species, such as Faecalibacterium prausnitzii, Prevotella copri, and Bacteroides vulgatus are shown and exhibited similar abundance in PAF and psAF but were remarkably distinct from non-AF controls (Fig. S3b, c, e, and f). Consistently, these observations were detected at the level of phylum (Fig. S1), class (Fig. S1), order (Fig. S2), family (Fig. S2), genus (Fig. S3), and species (Fig. S3).

10.1128/mSphere.00071-20.1 FIG S1 Taxonomic profiling at family level. (a and d) Venn diagrams demonstrating the number of annotated phyla (a) and classes (d) shared between non-AF control (CTR, blue), paroxysmal atrial fibrillation (PAF, yellow), and persistent atrial fibrillation (psAF, orange). The overlap shows that there were 103 phyla and 90 classes concurrently identified in PAF and psAF compared with non-AF CTR. (b and e) Bar plot of relative abundance of top-10 phyla (b) and classes (e) annotated in non-AF CTR (blue), PAF (yellow), and psAF (orange), where different taxa are differentiated by color. (c and f) Bar plot of relative abundance of top-10 phyla (c) and classes (f) annotated in individuals from non-AF CTR (blue), PAF (yellow), and psAF (orange), where different taxa are differentiated by color. Download FIG S1, PDF file, 1.5 MB. Copyright © 2020 Zuo et al. 2020 Zuo et al. This content is distributed under the terms of the Creative Commons Attribution 4.0 International license.

10.1128/mSphere.00071-20.2 FIG S2 Taxonomic profiling at genus level. (a and d) Venn diagrams demonstrating the number of annotated orders (a) and families (d) shared between non-AF control (CTR, blue), paroxysmal atrial fibrillation (PAF, yellow), and persistent atrial fibrillation (psAF, orange). The overlap shows that there were 181 orders and 368 families concurrently identified in PAF and psAF compared with non-AF CTR. (b and e) Bar plot of relative abundance of top-10 orders (b) and families (e) annotated in non-AF CTR (blue), PAF (yellow), and psAF (orange), where different taxa are differentiated by color. (c and f) Bar plot of relative abundance of top-10 orders (c) and families (f) annotated in individuals from non-AF CTR (blue), PAF (yellow), and psAF (orange), where different taxa are differentiated by color. Download FIG S2, PDF file, 1.5 MB. Copyright © 2020 Zuo et al. 2020 Zuo et al. This content is distributed under the terms of the Creative Commons Attribution 4.0 International license.

10.1128/mSphere.00071-20.3 FIG S3 Taxonomic profiling at species level. (a and d) Venn diagrams demonstrating the number of annotated genera (a) and species (d) shared between non-AF control (CTR, blue), paroxysmal atrial fibrillation (PAF, yellow), and persistent atrial fibrillation (psAF, orange). The overlap shows that there were 1,258 genera and 5,187 species concurrently identified in PAF and psAF compared with non-AF CTR. (b and e) Bar plot of relative abundance of top-10 genera (b) and species (e) annotated in non-AF CTR (blue), PAF (yellow), and psAF (orange), where different taxa are differentiated by color. (c and f) Bar plot of relative abundance of top-10 genera (c) and species (f) annotated in individuals from non-AF CTR (blue), PAF (yellow), and psAF (orange), where different taxa are differentiated by color. Download FIG S3, PDF file, 0.9 MB. Copyright © 2020 Zuo et al. 2020 Zuo et al. This content is distributed under the terms of the Creative Commons Attribution 4.0 International license.

In our current cohort, PAF and psAF patients were older, with higher BMI and lower TC and comorbid with T2DM compared with non-AF controls. As aging, higher BMI, HTN, and T2DM are risk factors for AF, the population bias should be considered indeed. Aiming to address whether our results of gut microbiota analysis were influenced by these risk factors, a multivariate linear regression analysis adjusting for age, BMI, HTN, T2DM, and TC was performed. Also, the independent strength of association between AF and the GM signatures, including alpha and beta diversity at the genus and species level, was examined. The results showed that AF was associated with increased microbial gene number, Shannon index, Pielou evenness, Chao richness, altered PCA, PCoA, NMDS index, and *Firmicutes*/*Bacteroidetes* (F/B) ratio as well as higher abundance of *Firmicutes* and lower abundance of *Bacteroidetes* independent of age, BMI, HTN, or T2DM (Table S2). We found that only the microbial PCA index at the genus level was associated with lower TC. This is consistent with previous reports indicating an association between disordered GM and lipid metabolic disturbance (22–24). We further compared the beta indices in the linear regression, where the beta index for TC (0.232) was lower than for AF (0.386). Therefore, it was concluded that the contribution of TC to disordered GM was less than that of AF. Notably, T2DM was not associated with microbial diversity or F/B ratio, with relatively low beta index and high *P* values.

10.1128/mSphere.00071-20.9 TABLE S2 Multivariate linear regression shows the independent strength of association between AF and GM signatures. Download Table S2, DOCX file, 0.02 MB. Copyright © 2020 Zuo et al. 2020 Zuo et al. This content is distributed under the terms of the Creative Commons Attribution 4.0 International license.

**FIG 2:**
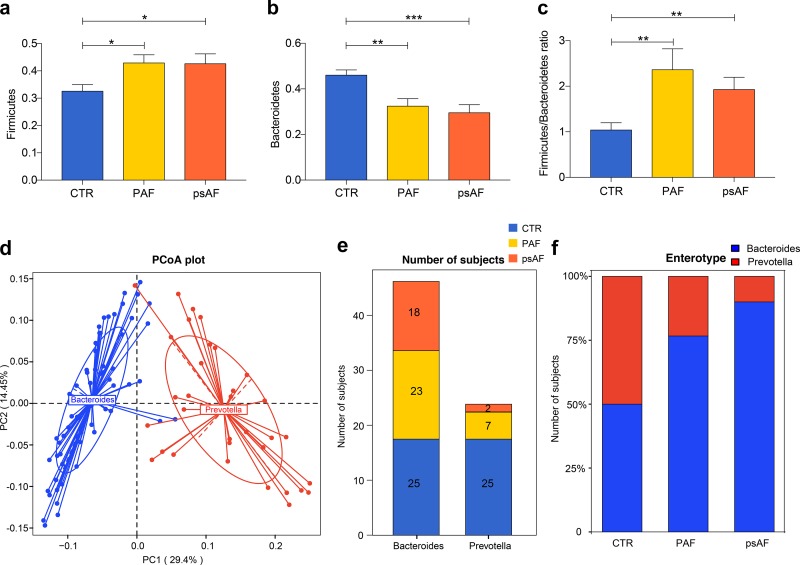
Similar community types between PAF and psAF. (a to c) Bar plot of *Firmicutes* (a), *Bacteroidetes* (b), and *Firmicutes*/*Bacteroidetes* ratio (c) in the non-AF control (CTR, blue), paroxysmal atrial fibrillation (PAF, yellow), and persistent atrial fibrillation (psAF, orange). *P* value: CTR versus PAF, *P* = 0.011 for *Firmicutes*, *P* = 0.002 for *Bacteroidetes*, *P* = 0.002 for *Firmicutes*/*Bacteroidetes* ratio; CTR versus psAF, *P* = 0.025 for *Firmicutes*, *P* < 0.001 for *Bacteroidetes*, *P* = 0.001 for *Firmicutes*/*Bacteroidetes* ratio; PAF versus psAF, *P* = 0.968 for *Firmicutes*, *P* = 0.621 for *Bacteroidetes*, *P* = 0.707 for *Firmicutes*/*Bacteroidetes* ratio. *, *P* < 0.05; **, *P* < 0.01; ***, *P* < 0.001. All data represent mean ± SEM. (d) One hundred samples are clustered into two enterotypes, and the major contributor in the two enterotypes is *Bacteroides* (blue) and *Prevotella* (red) by principal-component analysis of Jensen-Shannon divergence values at the genus level. (e and f) The number of subjects (e) and the percentage of control and AF samples (f) distributed in enterotype *Bacteroides* and enterotype *Prevotella*. There were 50% controls in enterotype *Bacteroides* and 50% controls in enterotype *Prevotella*, 76.67% PAF and 90% psAF participants in enterotype *Bacteroides*, and 23.33% PAF and 10% psAF participants in enterotype *Prevotella*. *P* = 0.0207, control versus PAF; *P* = 0.0023, control versus psAF; *P* = 0.2874, PAF versus psAF; Fisher’s exact test.

The shift of GM community structures in PAF and psAF subjects was investigated, and the ratio of *Firmicutes* and *Bacteroidetes* (F/B ratio) and microbial enterotype features were examined. The relative abundance of *Firmicutes* (*P* = 0.968, Fig. 2a), *Bacteroidetes* (*P* = 0.621, Fig. 2b), and F/B ratio (*P* = 0.707, Fig. 2c) were quite similar in PAF and psAF but were significantly different from non-AF controls (*Firmicutes*: *P* = 0.011, PAF versus CTR; *P* = 0.025, psAF versus CTR; *Bacteroidetes*: *P* = 0.002, PAF versus CTR; *P* < 0.001, psAF versus CTR; F/B ratio: *P* = 0.002, PAF versus CTR; *P* = 0.001, psAF versus CTR). Then, the 100 samples were divided into two enterotypes using PCA based on the Jensen-Shannon divergence (Fig. 2d). *Bacteroides* and *Prevotella* were the dominant bacteria in the observed enterotypes, respectively (Fig. 2d). Interestingly, there was a similar trend of enterotype distribution in PAF and psAF groups, with a higher percentage in enterotype *Bacteroides* (*P* = 0.2847, Fisher’s exact test, Fig. 2e and f). Therefore, different types of AF share similar dysbiotic GM profiles.

### Similar profiles of differential bacteria and microbial functions in PAF and psAF.

**FIG 3:**
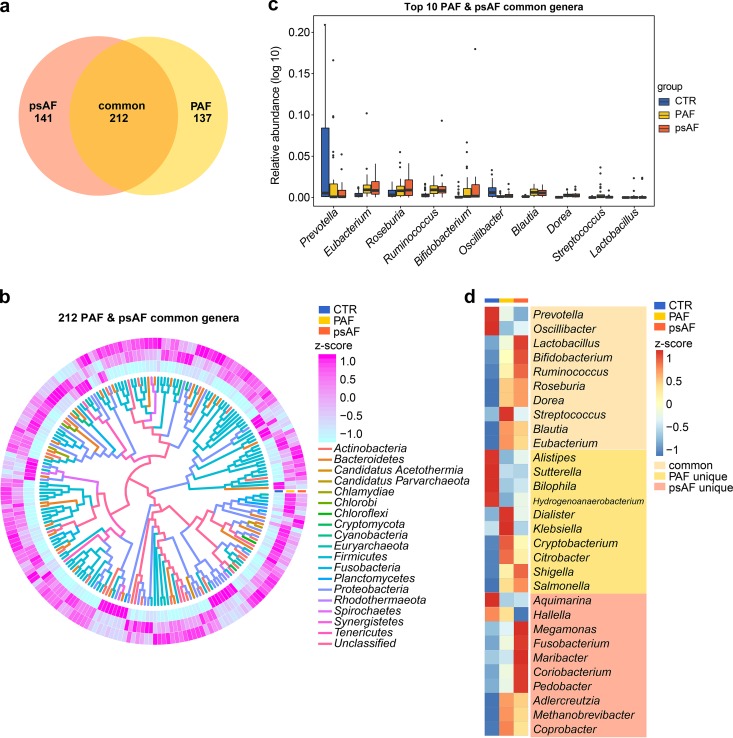
Similar profiles of differential genera in PAF and psAF. (a) Venn diagrams demonstrating the number of common differential genera shared between the paroxysmal atrial fibrillation (PAF, yellow) and persistent atrial fibrillation (psAF) groups compared to the non-AF control (CTR). The overlap shows that 212 genera were concurrently identified in PAF and psAF individuals. (b) Heatmap tree showing the 212 common differential genera in individuals from the PAF and psAF groups compared to the non-AF CTR at the criterion of *q* value of <0.05 (Wilcoxon rank sum test) and their phylogenic relationships. The abundance profiles are expressed by Z-scores, and genera were clustered based on Bray-Curtis distance in the clustering tree. The Z-score is negative (shown in blue) when the row abundance is lower than the mean and positive (pink) when the row abundance is higher than the mean. The color of the inner lines denotes the phyla of certain genera. (c) Box plot of top-10 common differential genera in individuals from the PAF (yellow) and psAF (orange) groups compared to the non-AF CTR (blue). Boxes represent the interquartile ranges, lines inside the boxes denote medians, and circles are outliers. (d) Heatmap of relative abundance of the top-10 genera common, unique to PAF, and unique to psAF at the criterion of *q* value of <0.05 (Wilcoxon rank sum test). The abundance profiles were transformed into Z-scores by subtracting the average abundance and dividing the standard deviation of all samples. The Z-score is negative (shown in blue) when the row abundance is lower than the mean and positive (red) when the row abundance is higher than the mean.

Subsequently, we analyzed the microbes that were dramatically differentially enriched among groups (*P* < 0.05; *P* values were tested using the Wilcoxon rank sum test and corrected for multiple testing with the Benjamini and Hochberg method). Compared with controls, 89 families, 349 genera, and 1,735 species were statistically different in PAF, and 105 families, 353 genera, and 1,742 species were statistically different in psAF. PAF and psAF shared a large number of these differentially enriched bacteria, including 53 families, 212 genera, and 1,123 species (Fig. 3a, Fig. S4a, and Fig. S5a). Notably, the majority of differentially enriched microbes were common to PAF and psAF and showed similar alterations (Fig. 3b to d, Fig. S4b to d, and Fig. S5b to d). It is therefore concluded that PAF and psAF exhibited a similar pattern of gut microbial features.

10.1128/mSphere.00071-20.4 FIG S4 Common differential families in PAF and psAF. (a) Venn diagrams demonstrating the number of common differential families shared between the paroxysmal atrial fibrillation (PAF, yellow) and persistent atrial fibrillation (psAF) groups compared to the non-AF control (CTR). The overlap shows that there were 53 families concurrently identified in PAF and psAF individuals. (b) Heatmap tree showing the 53 common differential families in individuals from the PAF and psAF groups compared to the non-AF CTR at the criterion of a *q* value of <0.05 (Wilcoxon rank sum test) and their phylogenic relationships. The abundance profiles are expressed by Z-scores, and genera were clustered based on Bray-Curtis distance in the clustering tree. The Z-score is negative (shown in blue) when the row abundance is lower than the mean and positive (pink) when the row abundance is higher than the mean. The color of the inner lines denotes the phyla of certain genera. (c) Box plot of top-10 common differential families in individuals from PAF (yellow) and psAF (orange) compared to non-AF CTR (blue). Boxes represent the interquartile ranges, lines inside the boxes denote medians, and circles are outliers. (d) Heatmap of relative abundance of the top-10 families common, unique to PAF, and unique to psAF at the criterion of a *q* value of <0.05 (Wilcoxon rank sum test). The abundance profiles were transformed into Z-scores by subtracting the average abundance and dividing the standard deviation of all samples. The Z-score is negative (shown in blue) when the row abundance is lower than the mean and positive (red) when the row abundance is higher than the mean. Download FIG S4, PDF file, 0.6 MB. Copyright © 2020 Zuo et al. 2020 Zuo et al. This content is distributed under the terms of the Creative Commons Attribution 4.0 International license.

10.1128/mSphere.00071-20.5 FIG S5 Common differential species in PAF and psAF. (a) Venn diagrams demonstrating the number of common differential species shared between the paroxysmal atrial fibrillation (PAF, yellow) and persistent atrial fibrillation (psAF) groups compared to the non-AF control (CTR). The overlap shows that there were 1,123 families concurrently identified in PAF and psAF individuals. (b) Heatmap tree showing the 1,123 common differential species in individuals from the PAF and psAF groups compared to the non-AF CTR at the criterion of a *q* value of <0.05 (Wilcoxon rank sum test) and their phylogenic relationships. The abundance profiles are expressed by Z-scores, and genera were clustered based on Bray-Curtis distance in the clustering tree. The Z-score is negative (shown in blue) when the row abundance is lower than the mean and positive (pink) when the row abundance is higher than the mean. The color of the inner lines denotes the phyla of certain genera. (c) Box plot of top-10 common differential species in individuals from PAF (yellow) and psAF (orange) compared to non-AF CTR (blue). Boxes represent the interquartile ranges, lines inside the boxes denote medians, and circles are outliers. (d) Heatmap of relative abundance of the top-10 species common, unique to PAF, and unique to psAF at the criterion of a *q* value of <0.05 (Wilcoxon rank sum test). The abundance profiles were transformed into Z-scores by subtracting the average abundance and dividing the standard deviation of all samples. The Z-score is negative (shown in blue) when the row abundance is lower than the mean and positive (red) when the row abundance is higher than the mean. Download FIG S5, PDF file, 1.4 MB. Copyright © 2020 Zuo et al. 2020 Zuo et al. This content is distributed under the terms of the Creative Commons Attribution 4.0 International license.

**FIG 4:**
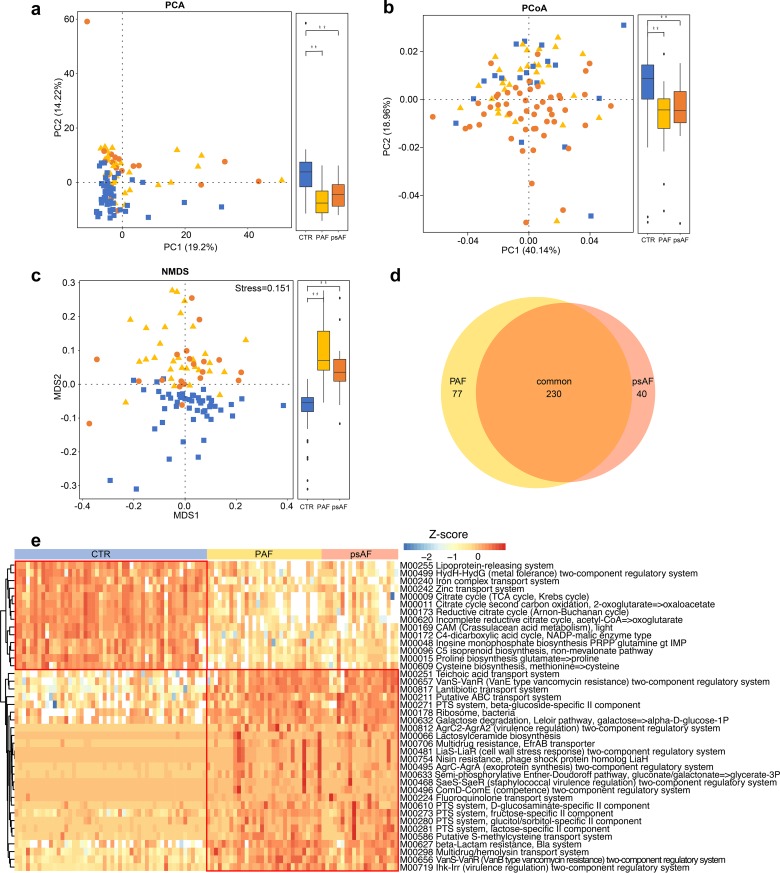
Similar profiles of differential microbial functions in PAF and psAF. (a to c) PCA (a), PCoA (b), and NMDS (c) based on abundances of the KEGG module showed disordered GM functional profiles in paroxysmal atrial fibrillation (PAF) and persistent atrial fibrillation (psAF). The blue squares represent non-AF control (CTR), yellow triangles refer to PAF, and orange circles denote psAF. (d) Venn diagram demonstrating the number of differential functional modules shared between PAF (yellow) and psAF (orange) compared with non-AF CTR. The overlap shows that there were 230 KEGG modules concurrently identified in PAF and psAF. (e) Heatmap of relative abundance of the 40 common functional modules at the criterion of *q* value of <0.0001 (Wilcoxon rank sum test). The abundance profiles are transformed into Z-scores by subtracting the average abundance and dividing the standard deviation of all samples. Z-score is negative (shown in blue) when the row abundance is lower than the mean and red when the row abundance is higher than the mean.

Furthermore, to depict the gut microbial gene functions in patients with PAF and psAF, the Kyoto Encyclopedia of Genes and Genomes (KEGG) databases were applied as we described previously (12). PCA, PCoA, and NMDS plots failed to distinguish PAF and psAF subjects but showed a clear separation between AF patients and non-AF CTRs (Fig. 4a to c). These data confirmed the similar alterations of GM between PAF and psAF in functions. Compared with the non-AF CTRs, there were 230 significantly different KEGG modules shared between the PAF and psAF groups (adjusted *P* < 0.05, Wilcoxon rank-sum test, Fig. 4d). The majority of these function modules shared the same trend in the PAF and psAF groups (Fig. 4e). The bacterial functions of the citric acid cycle and iron complex transport system, which are quite necessary for human physiological health, were deficient in the two AF groups (Fig. 4e). Moreover, six KEGG modules, such as pyrimidine deoxyribonucleotide biosynthesis and fatty acid biosynthesis, were significantly elevated in the psAF group compared to the PAF group (Fig. S6). The specific relationship of these gut microbial functions with the progression of AF remains to be elucidated.

10.1128/mSphere.00071-20.6 FIG S6 Distinctive KEGG modules between PAF and psAF. (a to f) Box plot of six distinctive KEGG modules between paroxysmal atrial fibrillation (PAF, yellow) and persistent atrial fibrillation (psAF) groups at the criterion of annotation in more than 10% of the samples. Boxes represent the interquartile ranges, lines inside the boxes denote medians, and circles are outliers. Download FIG S6, PDF file, 0.5 MB. Copyright © 2020 Zuo et al. 2020 Zuo et al. This content is distributed under the terms of the Creative Commons Attribution 4.0 International license.

### Similar metabolic features in PAF and psAF.

**FIG 5:**
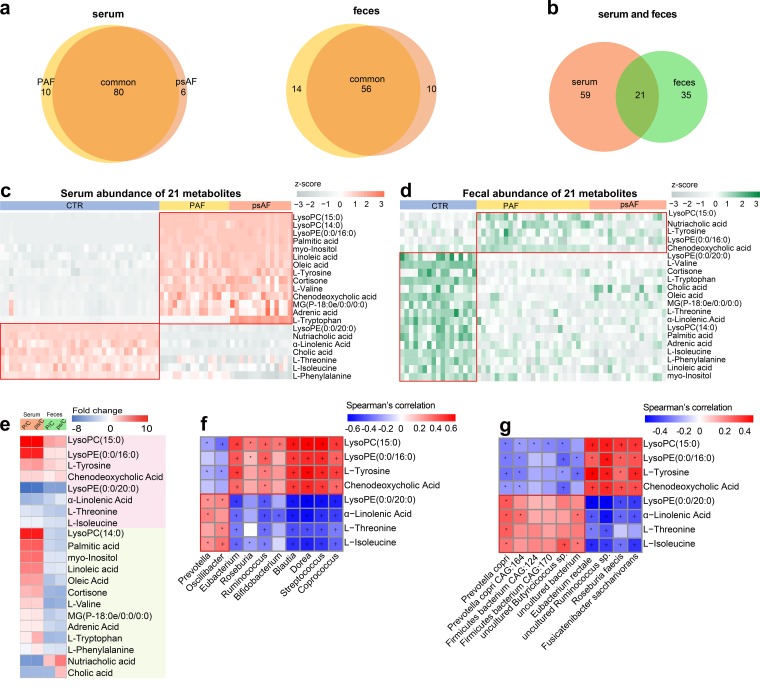
Similar metabolic features in PAF and psAF. (a and b) Venn diagrams demonstrating the number of differential metabolites shared between the paroxysmal atrial fibrillation (PAF, yellow) and persistent atrial fibrillation (psAF, orange) groups compared with the non-AF control (CTR). The overlap shows that there were 80 serum and 56 fecal metabolites concurrently identified in the non-AF and AF groups, while 21 endogenous compounds were concurrently identified in both feces and serum (b). (c and d) Heatmap of relative abundance of the 21 metabolites common to serum (c) and feces (d). The abundance profiles are transformed into Z-scores by subtracting the average abundance and dividing the standard deviation of all samples. The Z-score is negative (shown in gray) when the row abundance is lower than the mean and positive (orange for serum and green for feces) when the row abundance is higher than the mean. (e) Heatmap of fold change (AF/CTR) for 21 compounds that were altered in both serum and stool samples of AF patients. The fold change was transformed into t-scores, and the t-score is negative/positive (shown in blue/red) when the compound showed a decreased/increased tendency, respectively, in the PAF or psAF group. Compounds that increased or decreased simultaneously (*n* = 10) or individually (*n* = 11) in the serum and feces are shown in pink and green, respectively. (f and g) Relationship between 10 simultaneous metabolites and the top-10 common genera (f) and species (g). Considering the circulating metabolites that played a role during the process of GM-mediated responses, the serum data of metabolomic profiling were used for Spearman’s correlation analysis. Blue, negative correlation; red, positive correlation; *, *P* < 0.05; +, *P* < 0.01.

In addition, metabolomic analyses based on liquid chromatography-mass spectrometry (LC-MS) were performed to explore the metabolic profiles of PAF and psAF patients. A subset of 66 participants, consisting of 36 non-AF CTRs, 16 PAF patients, and 14 psAF patients from the present study, were included in the serum metabolic study, and 59 individuals consisting of 17 non-AF CTRs, 25 PAF patients, and 17 psAF patients were enrolled to examine the metabolomic profiles in the feces. For serum samples, 2,500 features at the positive ion mode (electrospray ionization positive [ESI^+^]) and 1,733 features at the negative ion mode (ESI^−^) were detected. For the fecal samples, 2,549 features at ESI^+^ and 1,894 features at ESI^−^ were observed. Generally, 80 serum metabolites and 56 fecal metabolites were found to be simultaneously altered in both PAF and psAF patients compared to the non-AF controls (Fig. 5a). There were 21 metabolites that overlapped in the serum and stool samples (Fig. 5b to d), eight of which were synchronously varied in the serum and feces (Fig. 5e and Table S3).

10.1128/mSphere.00071-20.10 TABLE S3 Detailed information for eight metabolites differently enriched across groups. Download Table S3, XLSX file, 0.01 MB. Copyright © 2020 Zuo et al. 2020 Zuo et al. This content is distributed under the terms of the Creative Commons Attribution 4.0 International license.

Correlation analyses between these 8 metabolites and the top-10 common genera (Fig. 5f) or species (Fig. 5g) in PAF and psAF were performed. The results showed that the PAF- and psAF-enriched metabolites, such as chenodeoxycholic acid (CDCA), were positively correlated with AF-enriched bacterial genera, including *Ruminococcus* and *Streptococcus*. *Ruminococcus* has been identified as a proinflammatory agent implicated in the development of inflammatory bowel disease (25). Transplantation of *Ruminococcus* into germfree mice has been reported to enhance the levels of gamma interferon, interleukin 17, and interleukin 22 (26). CDCA, a metabolite enriched in both PAF and psAF, plays an essential role in the progress of structural remodeling during AF (27). CDCA was found to be positively correlated with the left atrial low-voltage area (LVA) and to lead to apoptosis in atrial myocytes (27). The metabolites deficient in PAF and psAF, such as α-linolenic acid, were positively correlated with AF-decreased species, including Prevotella copri and Prevotella copri CAG:164. Previous reports have shown these species to be reduced in patients with Parkinson’s disease as well. These highly correlated linkages between specific taxa and metabolites were thus speculated to be the core features in PAF and psAF.

### Distinctive GM aspects between PAF and psAF.

**FIG 6:**
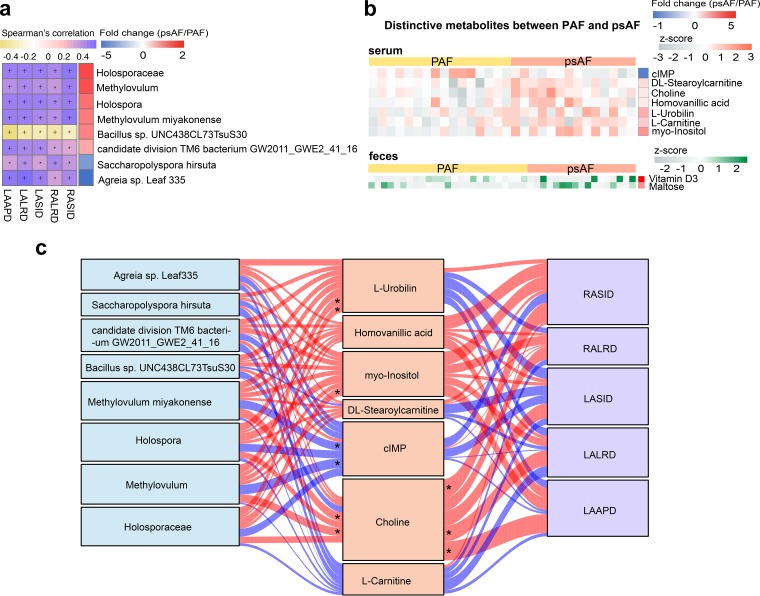
Correlation between distinctive taxa, microbial metabolites, and atrial diameter in PAF and psAF. (a) Spearman correlation between the 136 distinctive taxa between paroxysmal atrial fibrillation (PAF) and persistent atrial fibrillation (psAF) and atrial diameter, including left atrial anterior-posterior diameter (LAAPD), left atrial superior-inferior diameter (LASID), left atrial left-right diameter (LALRD), right atrial left-right diameter (RALRD), and right atrial superior-inferior diameter (RASID). The heatmap shows positive or negative correlations between distinctive taxa and atrial diameter, and the taxa described were correlated with five parameters of left and right atrial diameter with *P* < 0.05. Yellow, negative correlation; purple, positive correlation; *, *P* < 0.05; +, *P* < 0.01. The right column shows the heatmap of fold change (psAF/PAF) of 8 atrial diameter-correlated taxa. The fold change was transformed into t-scores, and the t-score is negative/positive (shown in blue/red) when the taxa showed a decreased/increased trend in the psAF group, respectively. (b) Heatmap of relative abundance of the 9 compounds in serum (*n* = 7) or feces (*n* = 2) that were distinctive between PAF and psAF. The abundance profiles are transformed into Z-scores by subtracting the average abundance and dividing the standard deviation of all samples. The Z-score is negative (shown in gray) when the row abundance is lower than the mean and positive (orange for serum and green for feces) when the row abundance is higher than the mean. The right column shows the heatmap of fold change (psAF/PAF) of 9 distinctive compounds. The fold change was transformed into t-scores, and the t-score is negative/positive (shown in blue/red) when the compound showed a decreased/increased trend in the psAF group, respectively. (c) Interrelationship between gut microbiota (GM) composition, host metabolic profile, and extent of atrial remodeling. Visualization of the correlation network according to Spearman correlation analysis between the distinctive taxa and the atrial diameter indicates how atrial remodeling was mediated by serum metabolites. Red connections indicate a positive correlation, while blue connections show correlations that were negative. LAAPD, left atrial anterior-posterior diameter; LASID, left atrial superior-inferior diameter; LALRD, left atrial left-right diameter; RALRD, right atrial left-right diameter; RASID, right atrial superior-inferior diameter.

Despite the similarity between GMs in PAF and psAF patients, some minor differences could be identified, with 8 families, 10 genera, and 118 species differentially enriched in PAF versus psAF (Fig. S7). To identify the most distinctive and functionally important taxa between PAF and psAF, correlation analyses of taxa and atrial diameter were performed. An enlarged atrium, including left atrial anterior-posterior diameter (LAAPD), left atrial superior-inferior diameter (LASID), left atrial left-right diameter (LALRD), right atrial left-right diameter (RALRD), and right atrial superior-inferior diameter (RASID), is the marker of irreversible atrial remodeling (28–31). Overall, one family (*Holosporaceae*), two genera (*Methylovulum* and *Holospora*), and five species including Methylovulum miyakonense, *Bacillus* sp. strain UNC438CL73TsuS30, candidate division TM6 bacterium GW2011_GWE2_41_16, Saccharopolyspora hirsuta, and *Agreia* sp. strain Leaf 335 were significantly correlated with the atrial diameter parameters (Fig. 6a).

10.1128/mSphere.00071-20.7 FIG S7 Distinctive taxa between PAF and psAF. Heatmap of relative abundance of the distinctive taxa between individuals from paroxysmal atrial fibrillation (PAF) and persistent atrial fibrillation (psAF), including 8 families (a), 4 genera (b), and 28 species (c) at the criterion of a *q* value of <0.05 (Wilcoxon rank sum test after filtering in less than 10% of the samples detected). The abundance profiles are transformed into Z-scores by subtracting the average abundance and dividing the standard deviation of all samples. Z-score is negative (shown in blue) when the row abundance is lower than the mean and red when the row abundance is higher than the mean. Download FIG S7, PDF file, 0.6 MB. Copyright © 2020 Zuo et al. 2020 Zuo et al. This content is distributed under the terms of the Creative Commons Attribution 4.0 International license.

The potential mechanisms mediating gut microbial function in human health rely on the interaction between gut microbe-derived metabolites and target organs (32, 33). Therefore, we focused on the 9 distinctive metabolites that differed between PAF and psAF (Fig. 6b), including serum myoinositol and fecal vitamin D_3_. To explore the association between AF severity and disordered gut microflora, we carried out correlation analysis between the distinctive taxa, metabolites, and atrial diameters (Fig. 6c). We found that psAF-enriched family *Holosporaceae* and genus *Holospora*, as well as genus *Methylovulum* and species Methylovulum miyakonense, were positively correlated with psAF-enriched serum choline. In addition, choline was significantly associated with enlarged atrium, the marker of irreversible structural remodeling in AF patients. The close relationship between gut microbes and left atrial diameter indicates that specific microbes might participate in the metabolism of specific metabolites and promote AF progression, which needs further investigation.

## DISCUSSION

In the present study, we have acquired evidence suggesting that different types of AF (PAF and psAF) show limited patterns of GM dysbiosis and alterations in metabolic features. Our previous study (20) identified the disordered GM profile in AF patients, but an understanding of the GM characteristics among heterogeneous AF individuals was still lacking. Therefore, we analyzed the similarity and disparity of GM profiles in PAF and psAF patients. We delineated a similarly increased microbial diversity in PAF and psAF, and the bacteria common to or uniquely enriched in PAF and psAF were identified. In addition, the close relationship between specific gut microbes, metabolites, and left atrial diameter revealed the potential role of gut bacteria in AF severity. These findings are fundamental for further studies exploring the key role of GM on AF progression.

One of the most important findings from the present study is that GM dysbiosis has already occurred in the self-terminating PAF. Studies focused on the dynamic correlation between gut microbiome and disease severity have shown similar phenomena in other diseases. These intriguing results point toward early alterations of GM in diseases and open the possibility for an important contribution by GM to disease pathogenesis. The specific gut bacteria and bioactive metabolites shared by PAF and psAF patients, such as *Ruminococcus* and CDCA, Prevotella copri, Prevotella copri CAG:164, and α-linolenic acid, are speculated to directly influence the progression of AF disease, or at least provide important biomarkers of AF.

Despite the great deal of similarity in GM between PAF and psAF patients, some GM species, like Methylovulum miyakonense, and certain metabolites like choline were distinct between PAF and psAF patients. The distinctive features of different AF types could be used as biomarkers to predict individuals with high risk of psAF. Certain AF-related GM features may be attractive candidates for detecting the disease at the self-terminating PAF. Moreover, many clinical studies have investigated the importance of choline as a biomarker for several human diseases, such as acute coronary syndrome (34–37). The significant association between GM, choline, and atrial diameter (RASLD, LALRD, and LAAPD) has been identified in the current work. It has been reported that choline could be produced through the lipid phosphatidylcholine metabolism by gut flora (38). Alterations in GM would lead to subsequent changes in choline production. Left atrial enlargement, reflected by atrial diameter, is the marker of irreversible atrial remodeling (28–31). Also, previous studies have shown that left atrial diameter is an independent risk factor for the development of AF and the long-term outcomes of ablation (39, 40). Therefore, choline is speculated to be a potential player mediating the impacts of GM dysbiosis on psAF development, at least in part. For the mechanisms behind this, evidence demonstrating a link between choline and AF is increasing. A previous cohort study demonstrated that plasma choline is associated with subsequent risk of AF (41), suggesting a potential role of choline metabolism in AF pathogenesis. Moreover, in canine and guinea pig atrial myocytes, choline has been shown to activate the acetylcholine-activated inward rectifier potassium current (I_KACh_), an outward K^+^ current, in a voltage-dependent manner (42). Constitutively, activation of the I_KACh_ is quite essential for cardiac electrical activity and AF pathophysiology. In psAF, the constitutively activated I_KACh_ was considered a background inward rectifier and therefore a contributor to shortened action potential duration and stable formation and high-frequency electrical rotors, leading to psAF (43, 44). Consequently, targeting choline to block I_KACh_ could potentially be antiarrhythmic in psAF (45, 46). In addition, a higher concentration of plasma choline was associated with lower high-density lipoprotein (HDL) cholesterol, an unfavorable cardiometabolic risk factor associated with psAF development (47, 48). Thus, GM and metabolites might act as a modulator of AF and may further be targets of preventive interventions for individuals at risk of AF. Even so, confirming the causal relationship between GM, choline, and psAF and exploring the precise underlying mechanisms remain to be further investigated.

AF is a clinically heterogeneous arrhythmia which is currently classified according to the manifestations of electrocardiogram (2). In most cases of AF, the disease progresses from low to heavy burden, advancing from short, infrequent episodes to longer and more frequent attacks (3, 4). Many AF patients do not receive therapy until the burden of disease progresses (49). However, AF duration is accompanied by irreversible structural injuries such as atrial remodeling and predicts a low sinus rhythm maintenance rate even after drug or ablation therapy (7, 8). AF-induced atrial remodeling enhances the vulnerability of the heart to AF induction and maintenance, with alterations in atrial refractivity, changes in cellular calcium homeostasis, autonomic activation, and afterdepolarizations, which contribute to triggered activity and AF initiation (50). Furthermore, structural remodeling dominated by atrial fibrosis leads to local conduction disturbances and blockages, which facilitates reentry and AF sustenance (51, 52). This autoreinforcing property of AF is often referred as “AF begets AF” (7, 53–55). Therefore, early intervention offers a crucial opportunity to halt the progressive pathoelectrophysiological and anatomical changes associated with AF. Our present findings provide further support for the importance of early intervention in AF and suggest that GM and corresponding metabolites might be potential therapeutic targets.

Consideration of possible limitations is necessary and can help to improve future studies. First, as AF is a progressive disease, collecting fecal samples from the patient cohort at different time points to longitudinally track the dynamic progress of disease may provide additional insight into GM patterns during AF. Further studies comparing how GM shifts from PAF to psAF might illuminate the interrelationship between AF phenotypes and GM. Second, although we excluded participants who used antibiotics or probiotics, exercise and dietary information were not collected and corrected. As well, a validation cohort is crucial for the confirmation of our results from the current discovery cohort. Third, the conclusions drawn from our data were associations rather than causal relationships. Further studies such as fecal microbiota transplantation and exploration for the effect of certain bacterial metabolites on electrophysiological modulation and AF onset are still needed. Finally, the metabolomics analysis was not performed for all the participants due to complex reasons in sample collection for some individuals. Also, as the metabolomic analysis was nontargeting LC-MS, the abundance of metabolite that we obtained was relative abundance. The present results provide preliminary clues and evidence for future investigations regarding the potential mechanisms between gut microbes and AF.

### Conclusions.

The present study provides a comprehensive description of the GM profiles of PAF and psAF patients and concludes that different types of AF show a limited degree of GM shift. GM dysbiosis has already occurred in mild stages of AF, which might act as an early modulator of disease and therefore may be regarded as a potential target to postpone AF progression.

## MATERIALS AND METHODS

### Study cohort.

Fifty nonvalvular AF patients and 50 matched controls (CTRs) were enrolled from our previous work (20). Nonvalvular AF patients with history of heart failure, coronary heart disease, structural heart disease, comorbidities (inflammatory bowel diseases, irritable bowel syndrome, autoimmune diseases, liver diseases, renal diseases, or cancer), or use of antibiotics or probiotics in the last 1 month were excluded. The clinical baseline characteristics were obtained via face-to-face surveys and medical records. The AF patients were divided into different groups based on AF history and manifestation of electrocardiogram. Paroxysmal AF (PAF) is characterized by self-termination, in most cases within 48 h, although some AF paroxysms may continue for up to 7 days, while persistent AF (psAF) is defined as AF that lasts longer than 7 days (2). The research protocol was approved by the ethics committee of Beijing Chaoyang Hospital and Kailuan General Hospital. All of the participants signed informed consent forms.

### Analyses of GM composition based on metagenome and metabolome.

The whole-metagenome shotgun sequencing data of the 100 feces samples in the current study were obtained as part of our previously published study (20). The analysis process of metagenomic sequencing, gene catalogue construction, gene prediction, taxonomic and functional annotation, abundance profiling, and analysis of enterotype were all performed as we previously described (20). In the 100 subjects enrolled in the current study, metabolomic data for 66 serum and 59 feces samples based on liquid chromatography-mass spectrometry (LC-MS) were available from our previous study (20). Methods of feature extraction, data normalization, and identification of compounds were carried out as we previously described (20). Compounds significantly distinguished between groups were identified by a variable influence on projection of >1 and *P* < 0.05 based on the peak areas.

### Statistical analysis.

Quantitative data with normal distributions are shown as mean ± standard deviation, while quantitative data with nonnormal distributions are presented as median (first quartile, third quartile), and the *t* test or Wilcoxon rank sum test was performed for between-group comparisons. Qualitative data are presented as a percentage, and the χ^2^ test was used for between-group comparisons. Pielou evenness, Shannon index, and Chao richness were calculated with R software (version 3.3.3, package vegan). Principal-component analysis (PCA) was performed by the FactoMineR package, principal-coordinate analysis (PCoA) was performed by the vegan and ape packages, and nonmetric dimensional scaling (NMDS) was performed by the vegan package, while all plots were visualized by the package ggplot2 in R software (version 3.3.3). Differential abundance of genes, genera, species, and KEGG modules was determined using the Wilcoxon rank sum test, and *P* values were corrected for multiple testing with the Benjamini and Hochberg method. The Spearman correlation of metabolic and microbiome abundances was used to identify microbiome-metabolome associations. All statistical tests were 2-sided, and *P* < 0.05 was regarded as significant.

### Data availability.

The data supporting the results of this article have been deposited in the EMBL European Nucleotide Archive (ENA) under the BioProject accession code PRJEB28384. The metabolomics data are available at the NIH Common Fund’s Data Repository and Coordinating Center website with Metabolomics Work-bench Study identifiers ST001168 (for fecal metabolomic analyses) and ST001169 (for serum metabolomic analyses).
