# Supplementary material for: Different Types of Atrial Fibrillation Share Patterns of Gut Microbiota Dysbiosis
Source: mSphere. 2020 Mar 18;5(2):e00071-20. doi: 10.1128/mSphere.00071-20 (PMC7082137; doi:10.1128/mSphere.00071-20)
Supplement: TABLE S1 [file mSphere.00071-20-st001.docx]

|  | **CTR** | **PAF** | **psAF** | **P value**  **(CTR vs. PAF)** | **P value**  **(CTR vs. psAF)** | **P value**  **(PAF vs. psAF)** |
| --- | --- | --- | --- | --- | --- | --- |
| **Number** | 50 | 30 | 20 | / | / | / |
| **Age, years** | 55 (50.5, 57.5) | 64 (51.75, 70) | 68 (61.25, 72.75) | <0.001 | <0.001 | 0.184 |
| **Male/ Female** | 41/9 | 17/13 | 28/8 | 0.015 | 0.511 | 0.190 |
| **BMI** | 24.77 (22.79, 27.62) | 25.71 (23.47, 27.83) | 27.34 (24.41, 31.24) | 0.440 | 0.028 | 0.120 |
| **HTN** | 27 | 16 | 11 | 0.954 | 0.940 | 0.909 |
| **DM** | 0 | 7 | 5 | <0.001 | <0.001 | 0.894 |
| **TC** | 4.73 (4.36, 5.33) | 4.35 (3.24, 4.73) | 4.09 (3.52, 4.57) | 0.004 | 0.001 | 0.866 |
| **LDL** | 2.3 (1.96, 2.86) | 2.45 (1.5, 3.03) | 2.45 (1.75, 2.88) | 0.948 | 0.912 | 0.968 |
| **FBG** | 5.12 (4.56, 5.55) | 4.95 (4.54, 5.79) | 4.96 (4.47, 5.86) | 0.541 | 0.830 | 0.859 |
| **Creatinine** | 70 (60, 89.5) | 63.8 (59.45, 82.78) | 71.85 (64.95, 77.6) | 0.218 | 0.662 | 0.148 |
| **ALT** | 19 (12, 25) | 19 (16, 31.25) | 21 (12.25, 28) | 0.191 | 0.431 | 0.728 |
| **ACEI** | 0 | 5 | 2 | / | / | / |
| **ARB** | 0 | 1 | 3 | / | / | / |
| **Amiodarone** | 0 | 6 | 3 | / | / | / |
| **Statin** | 0 | 2 | 2 | / | / | / |
| **DMBG** | 0 | 3 | 3 | / | / | / |

Table S1. Baseline clinical characteristics of the study cohort.

Abbreviations: AF, atrial fibrillation; BMI, body mass index; HTN, hypertension; DM, diabetes mellitus; CHD, coronary heart disease; TC, total cholesterol; TG, triglyceride; LDL, low density lipoprotein; FBG, fasting blood glucose; UA, uric acid; TBil, total bilirubin; ALT, glutamic-pyruvic transaminase; ACEI, angiotensin-converting enzyme inhibitors; ARB, angiotensin receptor blockers; DMBG, dimethyl biguanide; IQR, interquartile range; Data are presented as mean± SD, or median (IQR), as appropriate.
