# Supplementary material for: Different Types of Atrial Fibrillation Share Patterns of Gut Microbiota Dysbiosis
Source: mSphere. 2020 Mar 18;5(2):e00071-20. doi: 10.1128/mSphere.00071-20 (PMC7082137; doi:10.1128/mSphere.00071-20)
Supplement: FIG S1 [file mSphere.00071-20-sf001.pdf]

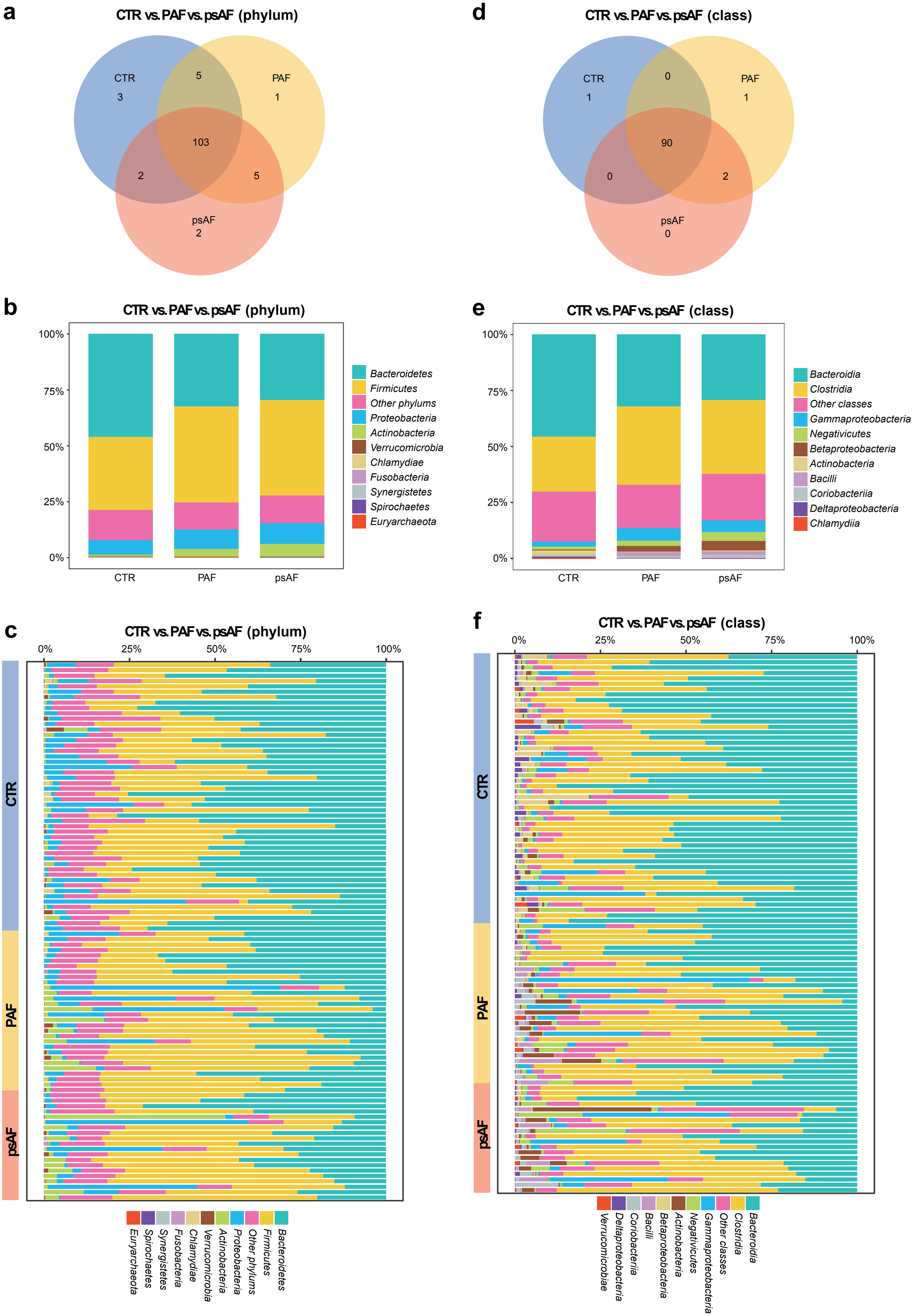

a
CTR vs. PAF vs. psAF (phylum)
CTR
3
5
PAF
1
103
2
5
psAF
2
d
CTR vs. PAF vs. psAF (class)
CTR
1
0
PAF
1
90
0
2
psAF
0
b
CTR vs. PAF vs. psAF (phylum)
100%
75%
50%
25%
0%
CTR
PAF
psAF
Bacteroidetes
Firmicutes
Other phylums
Proteobacteria
Actinobacteria
Verrucomicrobia
Chlamydiae
Fusobacteria
Synergistetes
Spirochaetes
Euryarchaeota
e
CTR vs. PAF vs. psAF (class)
Bacteroidia
Clostridia
Other classes
Gammaproteobacteria
Negativicutes
Betaproteobacteria
Actinobacteria
Bacilli
Coriobacteriia
Deltaproteobacteria
Chlamydiia
c
CTR vs. PAF vs. psAF (phylum)
0%
25%
50%
75%
100%
CTR
PAF
psAF
f
CTR vs. PAF vs. psAF (class)
Verrucomicrobiae
