# Supplementary figures and images for: Different Types of Atrial Fibrillation Share Patterns of Gut Microbiota Dysbiosis
Source: mSphere. 2020 Mar 18;5(2):e00071-20. doi: 10.1128/mSphere.00071-20 (PMC7082137; doi:10.1128/mSphere.00071-20)

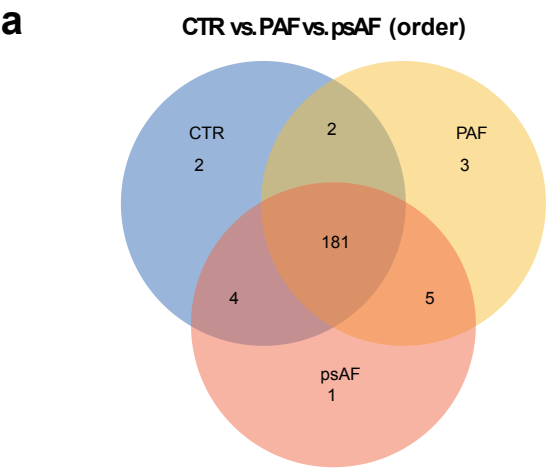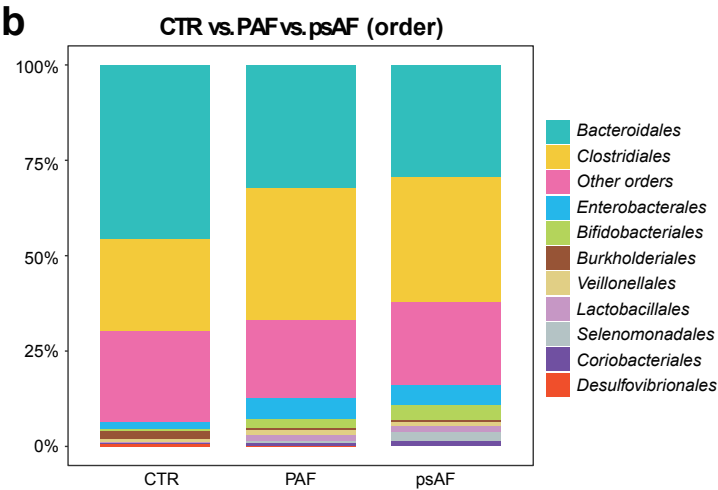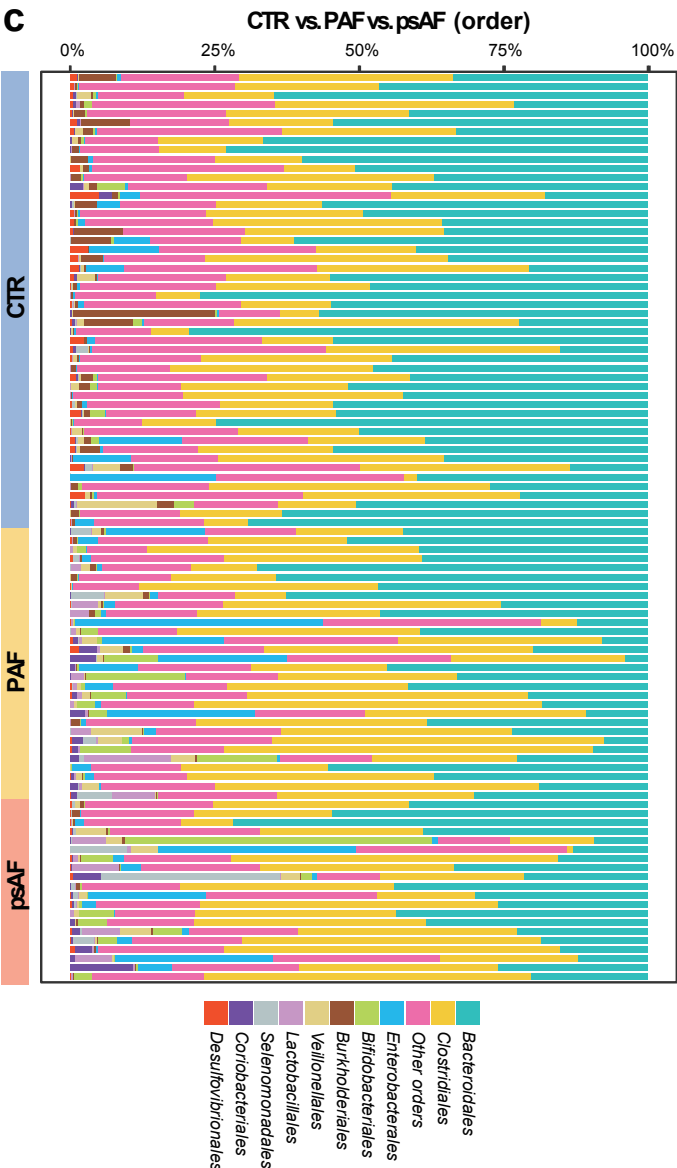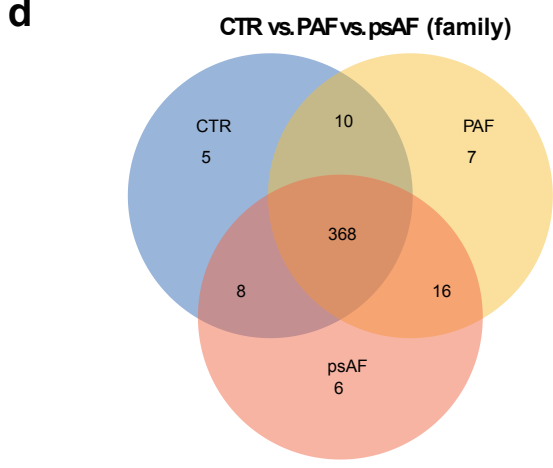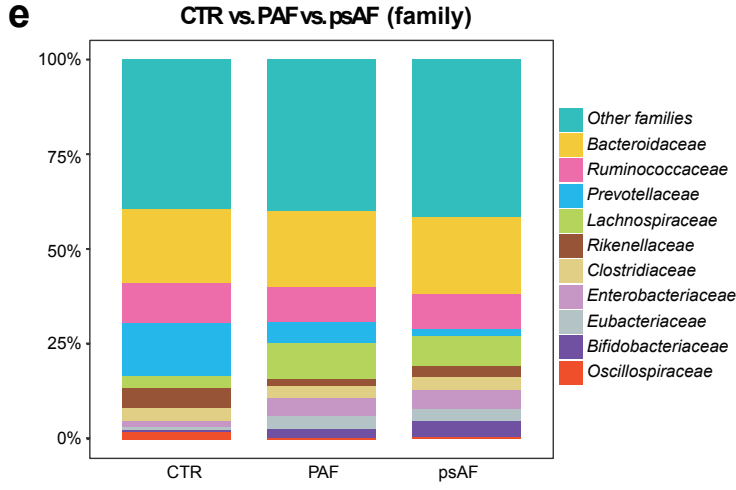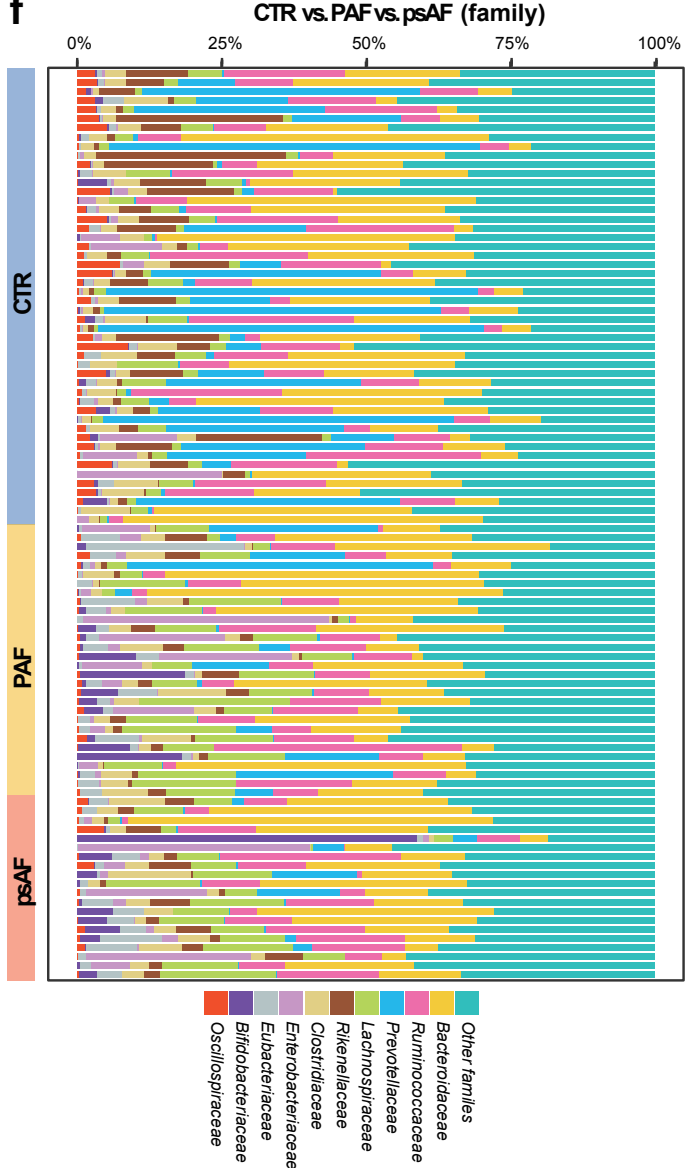

Supplement: FIG S2 [file mSphere.00071-20-sf002.pdf]

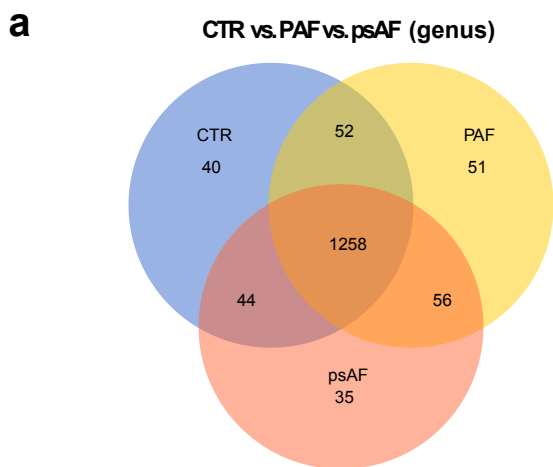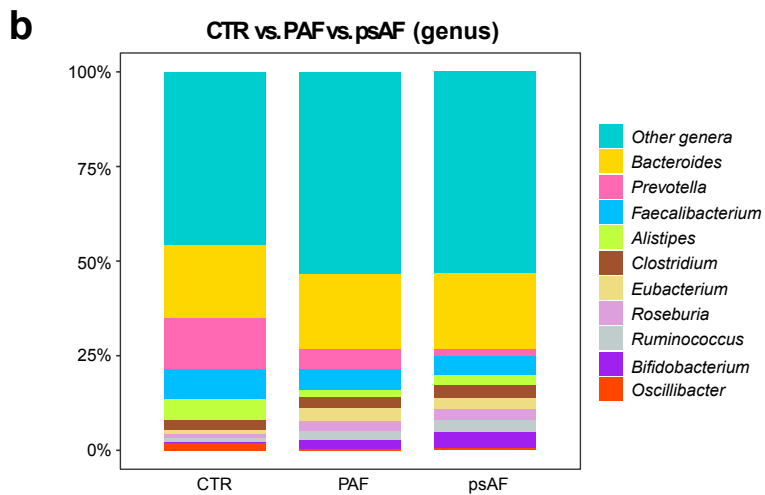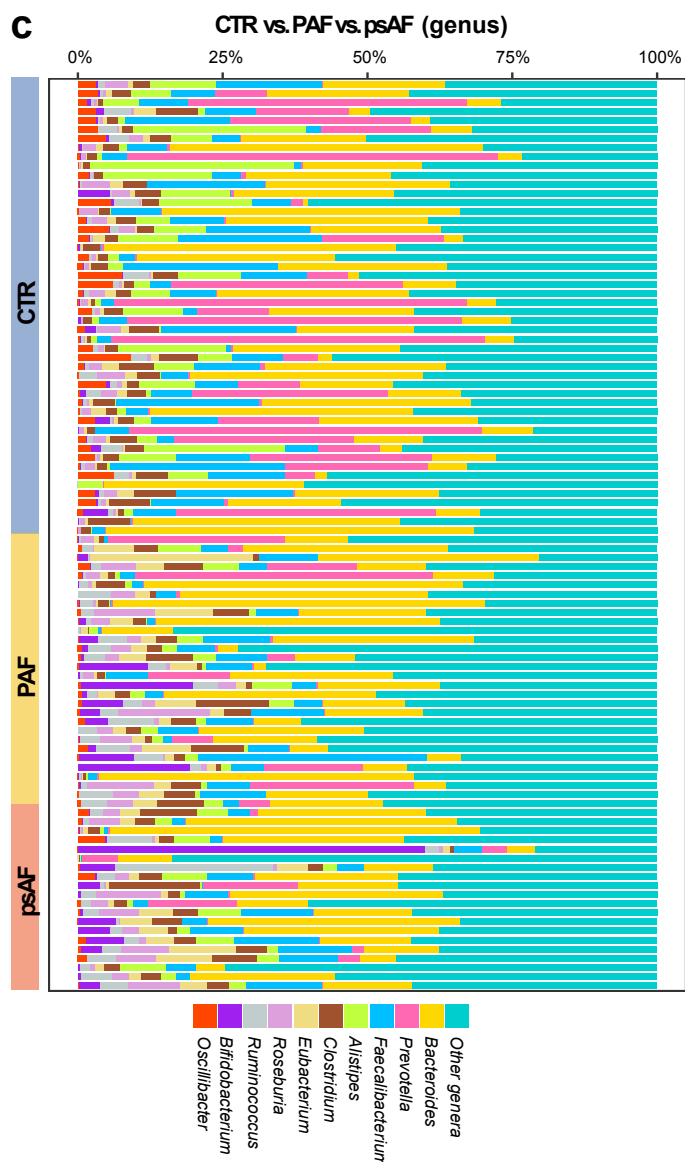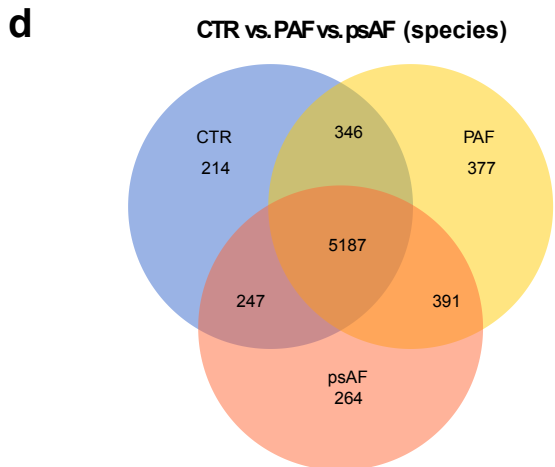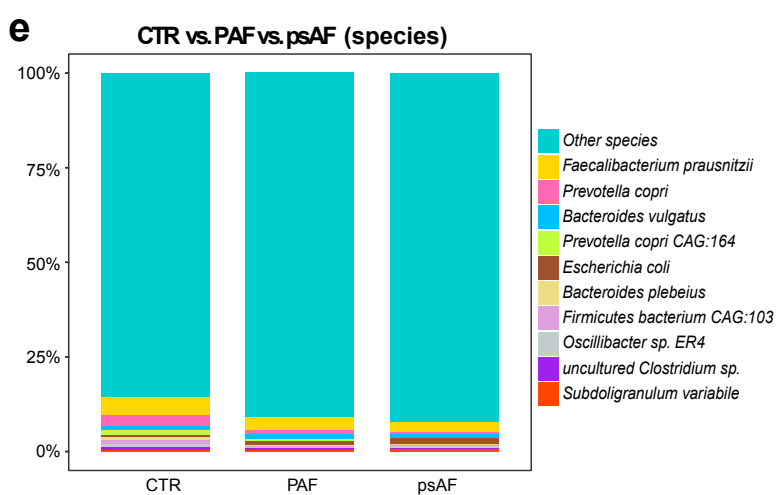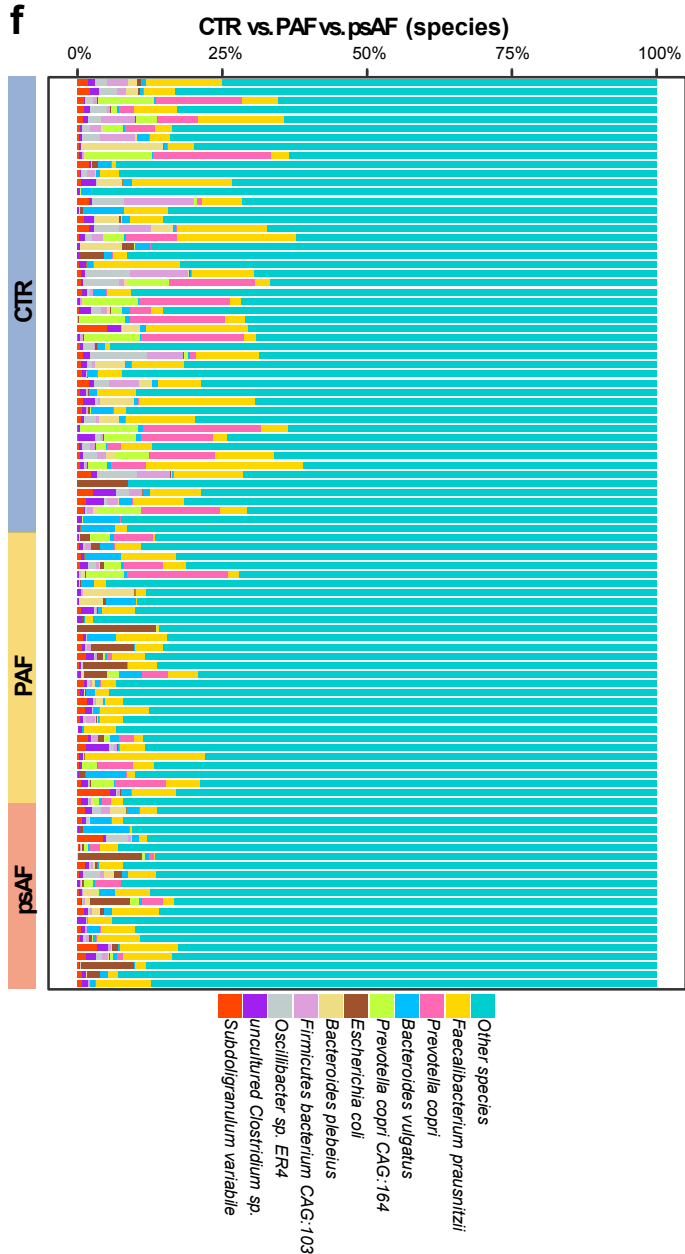

Supplement: FIG S3 [file mSphere.00071-20-sf003.pdf]

**a**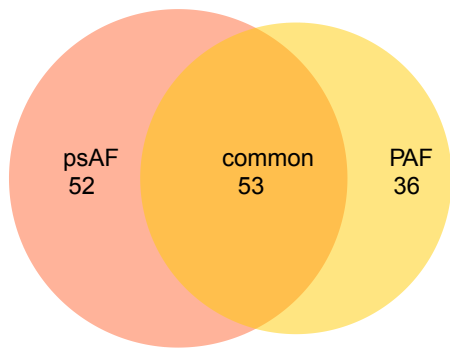**c**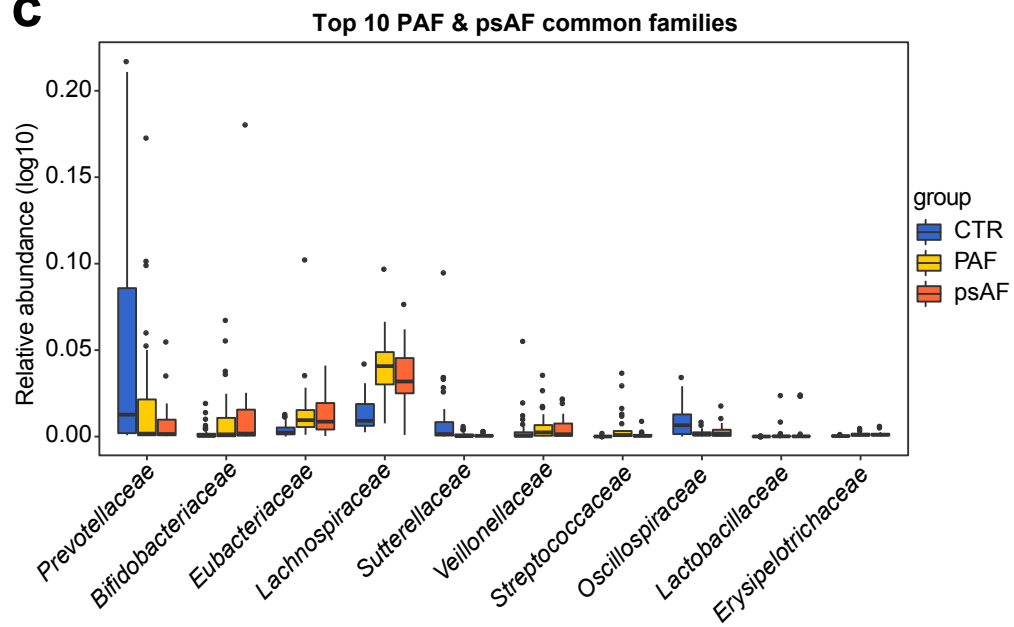**b****53 PAF & psAF common families**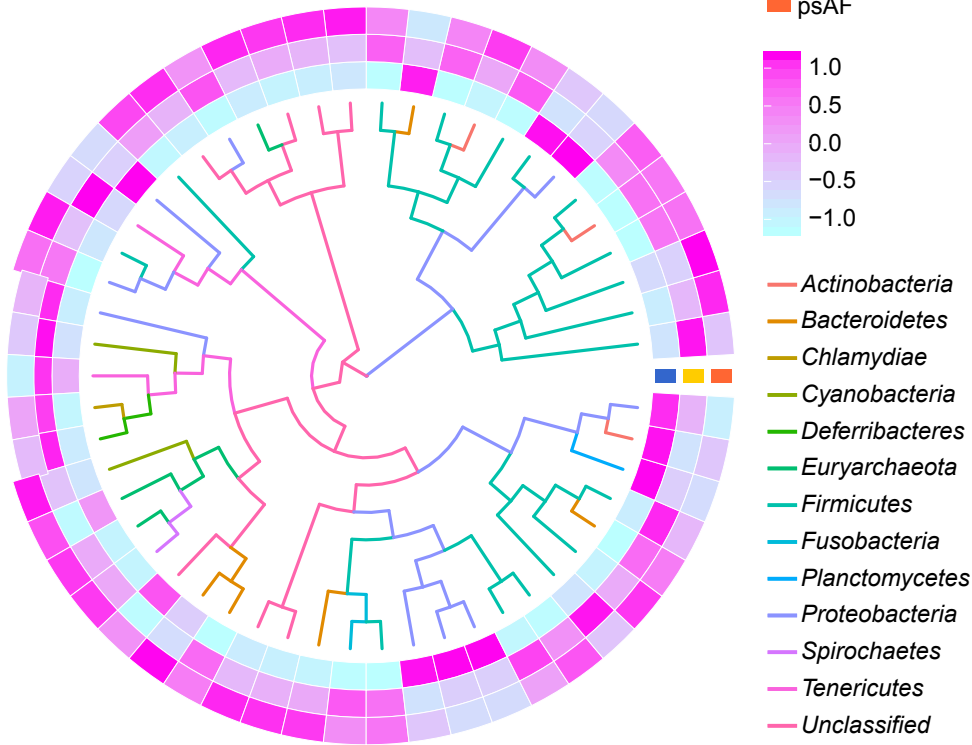**d**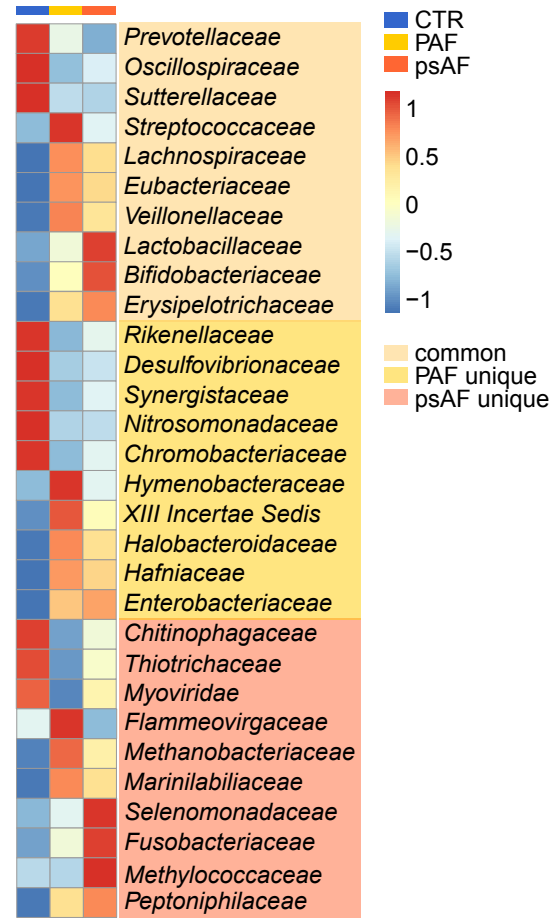

Supplement: FIG S4 [file mSphere.00071-20-sf004.pdf]

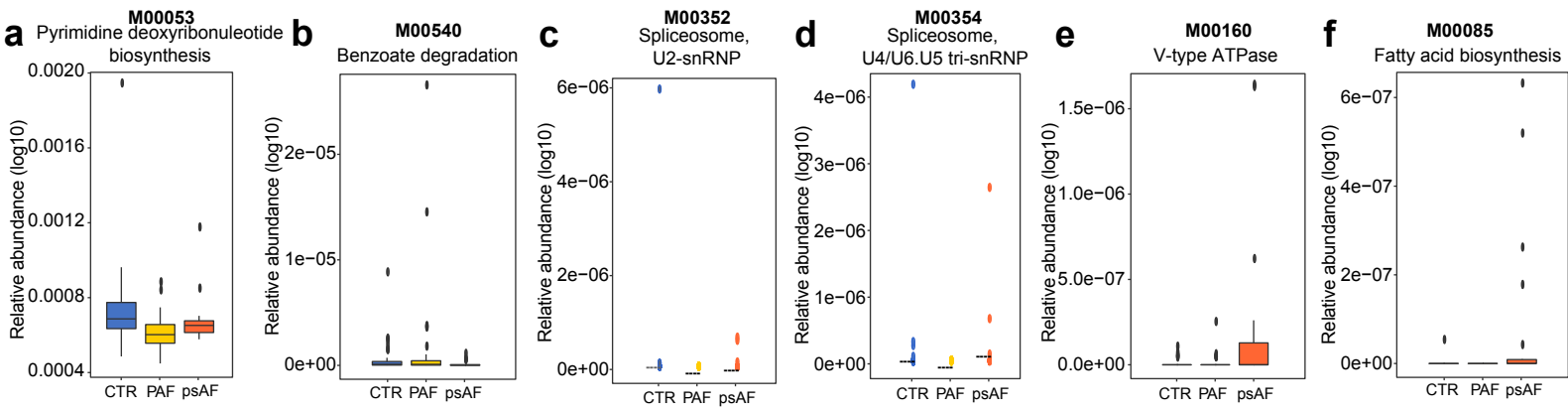

Supplement: FIG S6 [file mSphere.00071-20-sf006.pdf]

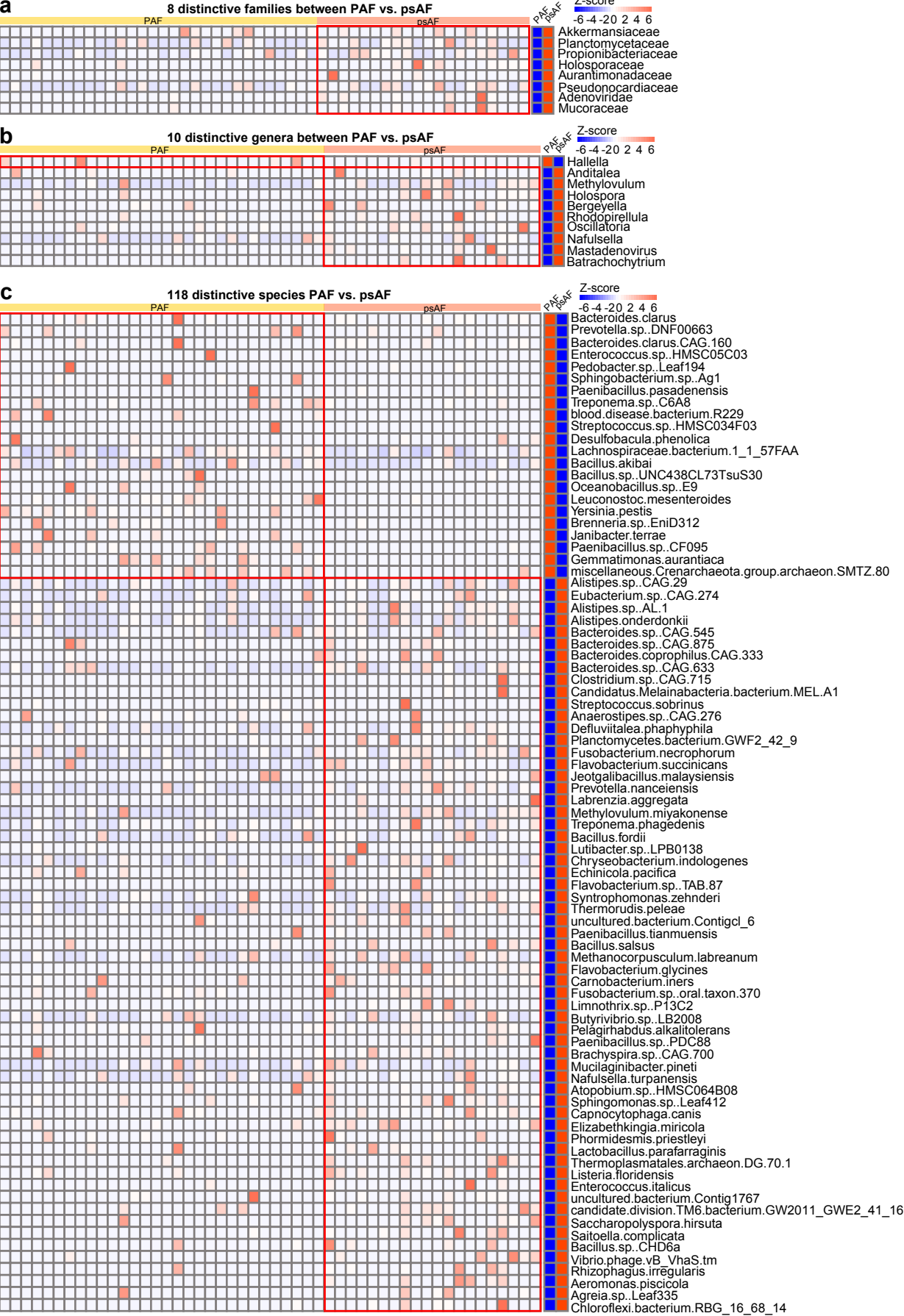

Supplement: FIG S7 [file mSphere.00071-20-sf007.pdf]
