# Supplementary material for: Different Types of Atrial Fibrillation Share Patterns of Gut Microbiota Dysbiosis
Source: mSphere. 2020 Mar 18;5(2):e00071-20. doi: 10.1128/mSphere.00071-20 (PMC7082137; doi:10.1128/mSphere.00071-20)
Supplement: TABLE S2 [file mSphere.00071-20-st002.docx]

**Table S2. Multivariate Linear Regression shows the independent strength of association between AF and GM signatures**

| **Alpha diversity** | **Gene Number** | | **Shannon index** | | | | **Chao richness** | | | | **Pielou evenness** | | | |
| --- | --- | --- | --- | --- | --- | --- | --- | --- | --- | --- | --- | --- | --- | --- |
|  |  |  | **genus** | | **species** | | **genus** | | **species** | | **genus** | | **species** | |
|  | ***Beta*** | ***p* value** | ***Beta*** | ***p* value** | ***Beta*** | ***p* value** | ***Beta*** | ***p* value** | ***Beta*** | ***p* value** | ***Beta*** | ***p* value** | ***Beta*** | ***p* value** |
| **AF** | 0.289 | **0.026** | 0.267 | **0.041** | 0.377 | **0.003** | 0.159 | 0.234 | 0.260 | **0.046** | 0.260 | **0.046** | 0.358 | **0.005** |
| **Age** | 0.034 | 0.780 | 0.091 | 0.462 | 0.034 | 0.770 | 0.058 | 0.646 | 0.060 | 0.625 | 0.101 | 0.410 | 0.021 | 0.857 |
| **BMI** | 0.071 | 0.482 | 0.026 | 0.801 | 0.127 | 0.191 | 0.055 | 0.601 | 0.057 | 0.580 | 0.021 | 0.839 | 0.133 | 0.174 |
| **TC** | -0.074 | 0.503 | 0.003 | 0.980 | -0.085 | 0.420 | -0.046 | 0.688 | -0.053 | 0.633 | 0.010 | 0.928 | -0.086 | 0.420 |
| **T2DM** | -0.187 | 0.081 | -0.005 | 0.960 | -0.139 | 0.172 | -0.183 | 0.099 | -0.206 | 0.057 | 0.032 | 0.763 | -0.104 | 0.309 |
| **HTN** | -0.102 | 0.335 | -0.021 | 0.846 | 0.023 | 0.821 | -0.059 | 0.590 | -0.069 | 0.521 | -0.017 | 0.869 | 0.046 | 0.649 |

| **Beta diversity** | **PCA** | | | | **PCoA** | | | | **NMDS** | | | |
| --- | --- | --- | --- | --- | --- | --- | --- | --- | --- | --- | --- | --- |
|  | **genus** | | **species** | | **genus** | | **species** | | **genus** | | **species** | |
|  | ***Beta*** | ***p* value** | ***Beta*** | ***p* value** | ***Beta*** | ***p* value** | ***Beta*** | ***p* value** | ***Beta*** | ***p* value** | ***Beta*** | ***p* value** |
| **AF** | 0.386 | **0.003** | 0.791 | **0.000** | 0.174 | 0.192 | -0.410 | **0.001** | 0.211 | 0.113 | 0.384 | **0.003** |
| **Age** | 0.079 | 0.508 | -0.113 | 0.205 | 0.132 | 0.298 | 0.109 | 0.359 | 0.094 | 0.458 | -0.138 | 0.249 |
| **BMI** | 0.047 | 0.634 | 0.052 | 0.483 | 0.050 | 0.632 | -0.058 | 0.560 | 0.066 | 0.529 | 0.058 | 0.558 |
| **TC** | 0.232 | **0.033** | -0.041 | 0.605 | 0.112 | 0.324 | 0.032 | 0.768 | 0.081 | 0.477 | -0.052 | 0.627 |
| **DM** | -0.039 | 0.704 | -0.113 | 0.145 | -0.027 | 0.805 | -0.052 | 0.616 | -0.053 | 0.628 | 0.076 | 0.463 |
| **HTN** | 0.041 | 0.690 | 0.014 | 0.857 | -0.020 | 0.855 | 0.005 | 0.964 | -0.058 | 0.596 | -0.024 | 0.816 |

| **F/B ratio** | **Firmicutes** | | **Bacteroidetes** | | **F/B ratio** | |
| --- | --- | --- | --- | --- | --- | --- |
|  | ***Beta*** | ***p* value** | ***Beta*** | ***p* value** | ***Beta*** | ***p* value** |
| **AF** | 0.294 | **0.026** | -0.371 | **0.003** | -0.292 | **0.024** |
| **Age** | 0.037 | 0.767 | -0.094 | 0.428 | -0.077 | 0.528 |
| **BMI** | -0.048 | 0.645 | -0.004 | 0.964 | 0.006 | 0.949 |
| **TC** | 0.043 | 0.702 | -0.107 | 0.316 | -0.017 | 0.875 |
| **DM** | 0.017 | 0.878 | -0.044 | 0.667 | -0.036 | 0.735 |
| **HTN** | 0.038 | 0.721 | 0.027 | 0.791 | 0.101 | 0.342 |

**Abbreviations**: AF, atrial fibrillation; GM, gut microbiota; BMI, body mass index; HTN, hypertension; DM, diabetes mellitus; TC, total cholesterol; HTN, hypertension; PCA, principal component analysis; PCoA, principal coordinates analysis; NMDS, non-metric dimensional scaling.
