# Supplementary material for: Different Types of Atrial Fibrillation Share Patterns of Gut Microbiota Dysbiosis
Source: mSphere. 2020 Mar 18;5(2):e00071-20. doi: 10.1128/mSphere.00071-20 (PMC7082137; doi:10.1128/mSphere.00071-20)
Supplement: FIG S5 [file mSphere.00071-20-sf005.pdf]

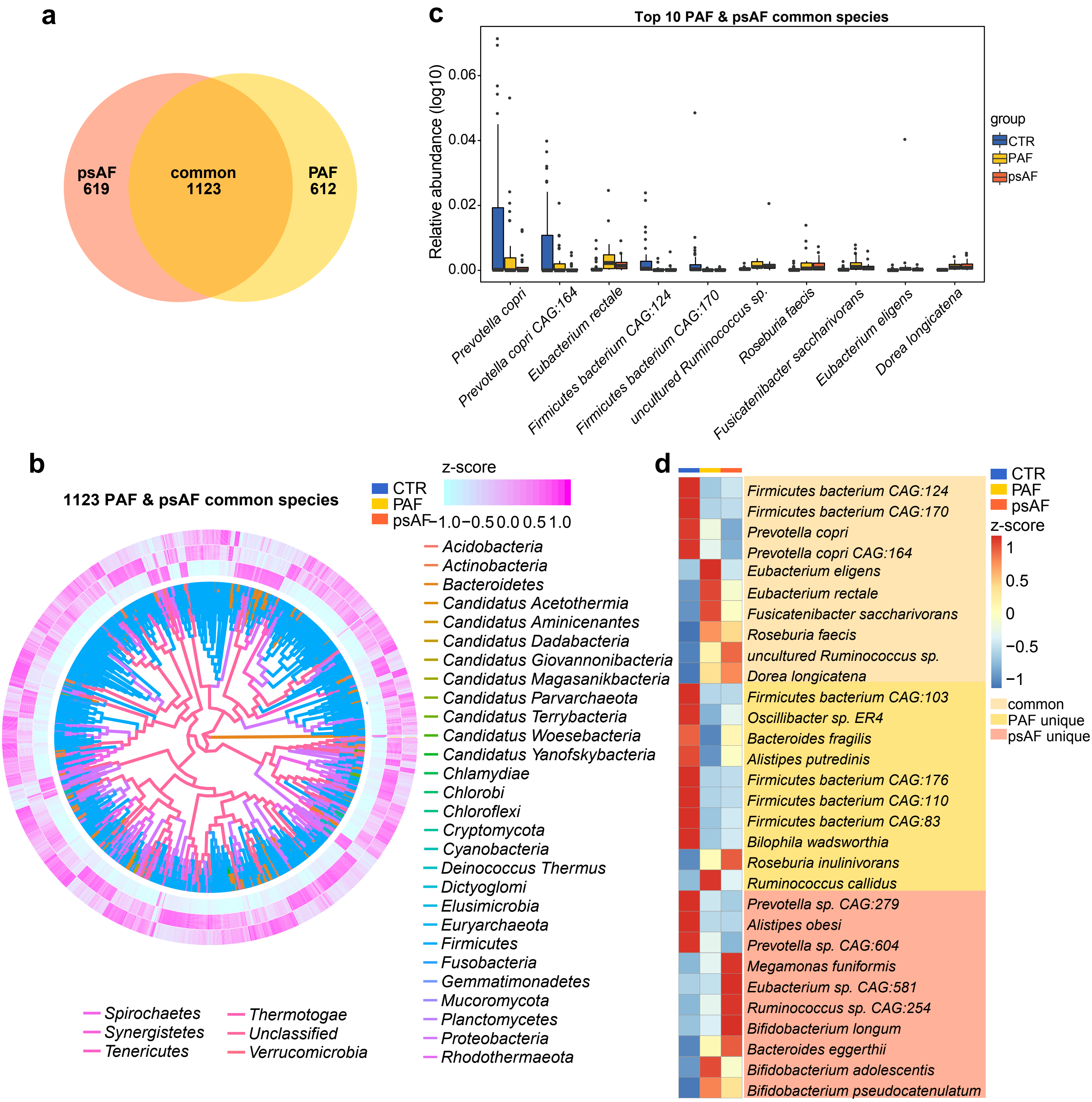
a
psAF
619
common
1123
PAF
612
c
Top 10 PAF & psAF common species
Relative abundance (log10)
0.06
0.04
0.02
0.00
group
CTR
PAF
psAF
Prevotella copri
Prevotella copri CAG:164
Eubacterium rectale
Firmicutes bacterium CAG:124
Firmicutes bacterium CAG:170
uncultured Ruminococcus sp.
Roseburia faecis
Fusicatenibacter saccharivorans
Eubacterium eligens
Dorea longicatena
b
1123 PAF & psAF common species
CTR
PAF
psAF
z-score
−1.0 −0.5 0.0 0.5 1.0
Acidobacteria
Actinobacteria
Bacteroidetes
Candidatus Acetothermia
Candidatus Aminicenantes
Candidatus Dadabacteria
Candidatus Giovannonibacteria
Candidatus Magasanikbacteria
Candidatus Parvarchaeota
Candidatus Terrybacteria
Candidatus Woesebacteria
Candidatus Yanofskybacteria
Chlamydiae
Chlorobi
Chloroflexi
Cryptomycota
Cyanobacteria
Deinococcus Thermus
Dictyoglomi
Elusimicrobia
Euryarchaeota
Firmicutes
Fusobacteria
Gemmatimonadetes
Mucoromycota
Planctomycetes
Proteobacteria
Rhodothermaeota
Spirochaetes
Synergistetes
Tenericutes
Thermotogae
Unclassified
Verrucomicrobia
d
Firmicutes bacterium CAG:124
Firmicutes bacterium CAG:170
Prevotella copri
Prevotella copri CAG:164
Eubacterium eligens
Eubacterium rectale
Fusicatenibacter saccharivorans
Roseburia faecis
uncultured Ruminococcus sp.
Dorea longicatena
Firmicutes bacterium CAG:103
Oscillibacter sp. ER4
Bacteroides fragilis
Alistipes putredinis
Firmicutes bacterium CAG:176
Firmicutes bacterium CAG:110
Firmicutes bacterium CAG:83
Bilophila wadsworthia
Roseburia inulinivorans
Ruminococcus callidus
Prevotella sp. CAG:279
Alistipes obesi
Prevotella sp. CAG:604
Megamonas funiformis
Eubacterium sp. CAG:581
Ruminococcus sp. CAG:254
Bifidobacterium longum
Bacteroides eggerthii
Bifidobacterium adolescentis
Bifidobacterium pseudocatenulatum
CTR
PAF
psAF
z-score
1
0.5
0
−0.5
−1
common
PAF unique
psAF unique
